# Histological and Top-Down Proteomic Analyses of the Visual Pathway in the Cuprizone Demyelination Model

**DOI:** 10.1007/s12031-022-01997-w

**Published:** 2022-05-30

**Authors:** Mohammed S. M. Almuslehi, Monokesh K. Sen, Peter J. Shortland, David A. Mahns, Jens R. Coorssen

**Affiliations:** 1 Department of Physiology & Pharmacology, College of Veterinary Medicine, University of Diyala, Baqubah, Diyala 32001 Iraq; 2 School of Medicine, Western Sydney University, Penrith, NSW 2751 Australia; 3 Charles Perkins Centre, School of Medical Sciences, University of Sydney, Camperdown, NSW 2006 Sydney, Australia; 4 School of Science, Western Sydney University, Penrith, NSW 2751 Australia; 5 Department of Integrative Physiology, School of Medicine, Western Sydney University, Locked Bag 1797, Penrith, NSW 2571 Australia; 6 Department of Health Sciences, Faculty of Applied Health Sciences, and Department of Biological Sciences, Faculty of Mathematics and Science, Brock University, ON L2S 3A1 St. Catharine’s, Canada

**Keywords:** Optic nerve, Optic tract, Optic neuritis, Multiple sclerosis, Proteoforms, Bioinformatics, 2D gel electrophoresis, Mass spectrometry

## Abstract

A change in visual perception is a frequent early symptom of multiple sclerosis (MS), the pathoaetiology of which remains unclear. Following a slow demyelination process caused by 12 weeks of low-dose (0.1%) cuprizone (CPZ) consumption, histology and proteomics were used to investigate components of the visual pathway in young adult mice. Histological investigation did not identify demyelination or gliosis in the optic tracts, pretectal nuclei, superior colliculi, lateral geniculate nuclei or visual cortices. However, top-down proteomic assessment of the optic nerve/tract revealed a significant change in the abundance of 34 spots in high-resolution two-dimensional (2D) gels. Subsequent liquid chromatography-tandem mass spectrometry (LC-TMS) analysis identified alterations in 75 proteoforms. Literature mining revealed the relevance of these proteoforms in terms of proteins previously implicated in animal models, eye diseases and human MS. Importantly, 24 proteoforms were not previously described in any animal models of MS, eye diseases or MS itself. Bioinformatic analysis indicated involvement of these proteoforms in cytoskeleton organization, metabolic dysregulation, protein aggregation and axonal support. Collectively, these results indicate that continuous CPZ-feeding, which evokes a slow demyelination, results in proteomic changes that precede any clear histological changes in the visual pathway and that these proteoforms may be potential early markers of degenerative demyelinating conditions.

**Supplementary Information:**

The online version contains supplementary material available at 10.1007/s12031-022-01997-w.

## Introduction

The optic nerve/tract is responsible for the relay of information from the retinal ganglion cells to the primary visual cortex via the lateral geniculate nucleus (Rizzo 2005; Selhorst and Chen 2009). Any insult to the optic pathway results in changes in visual perception (Athappilly et al. 2008; Dutton 2004; Kiyota et al. 2017). A common example of this is optic neuritis, a demyelinating and acute inflammatory disorder (Costello 2016; Kale 2016). Optic neuritis has been attributed to several aetiologies including genetics, inflammation, infections and exposure to toxic substances, and it is highly associated with multiple sclerosis (MS; Costello 2016; Kale 2016); however, the pathoaetiology of optic neuritis and MS-associated changes in vision is not clearly defined. It has been suggested that central nervous system (CNS) demyelination and inflammation are initiated in a slow and progressive manner that may start several years prior to the onset of clinical symptoms (Jones-Odeh and Hammond 2015; Olesen et al. 2019; Sen et al. 2020a; Stys et al. 2012). Moreover, changes in visual perception (e.g. blurring, eye fatigue, lack of sharpness and ocular pain) can also be seen in other neurodegenerative disorders such as Alzheimer’s and Parkinson’s diseases (Colligris et al. 2018; Kesler and Korczyn 2006). Like MS, in which the mechanism of visual disturbances is still unknown, the pathoaetiology of visual disturbances in Alzheimer’s and Parkinson’s has also not been identified.

These demyelination-related changes can be investigated using the cuprizone (CPZ) (bis(cyclohexanone)oxaldihydrazone) animal model (Sen et al. 2019b, 2020a). This model was initially used to study de- and re-myelination and the innate immune response (Goldberg et al. 2015; Kipp et al. 2009; Matsushima and Morell 2001; Praet et al. 2014; Sen et al. 2019b). More recently, the CPZ model has been modified to investigate adaptive immune responses in the CNS (Almuslehi et al. 2020; Caprariello et al. 2018; Sen et al. 2019a). In this model, animals are fed CPZ, which is preferentially toxic to mature oligodendrocytes, inducing oligodendrocytosis (i.e. loss or degeneration of oligodendrocytes) and subsequent demyelination, as well as micro- and astro-glial activation (Almuslehi et al. 2020; Sen et al. 2019a, 2020b, 2022). The mechanisms underlying oligodendrocytosis induced by CPZ are still poorly understood. It has been proposed that the metal (e.g. copper) chelating properties of CPZ disrupt metabolic processes in oligodendrocytes due to the inhibition of mitochondrial enzymes (reviewed in Sen et al. 2019b, 2022). However, very little is known about the actions of CPZ on the visual pathway, and much of what is reported is contradictory (reviewed in Sen et al. 2019b). One histological investigation found a reduction of myelin basic protein in optic nerves (Namekata et al. 2014), whereas no loss of myelin protein was observed in other studies (Araujo et al. 2017; Goldberg et al. 2015; Sen et al. 2020b; Yang et al. 2009). However, different experimental paradigms were used in these studies (e.g. dose, duration of CPZ-feeding and (immuno)histological staining techniques). Thus, feeding C57Bl/6 mice with 0.2% CPZ for 12 weeks led to a reduction of myelin basic protein in the optic nerve, which was unchanged in the first 6 weeks of CPZ-feeding (Namekata et al. 2014). Yet, feeding 0.2% CPZ for 3 or 5 weeks did not produce any changes in mRNA expression of myelin basic protein in optic nerve (Araujo et al. 2017). Another investigation showed no changes in myelin proteolipid protein when mice were fed with 0.25% CPZ for 5 weeks (Goldberg et al. 2015). Likewise, we showed that feeding 0.2% CPZ for 5 weeks did not result in loss of myelin in the optic nerve/tract when silver staining was used for myelin detection (Sen et al. 2020b). These findings indicate that changes in optic nerve/tract myelin status may rely on the dose of CPZ, duration of exposure or method used to assess myelin status. However, using an autoimmune animal model of (later stage) MS, ultrastructural analysis of optic nerves in mice subjected to experimental autoimmune encephalomyelitis (EAE) showed axonal demyelination relative to healthy control mice (Manogaran et al. 2019).

Apart from histological alterations, several studies have investigated proteome changes in the CNS of CPZ-fed mice (Martin et al. 2018; Partridge et al. 2016; Sen et al. 2019a; Szilagyi et al. 2020; Werner et al. 2010). However, proteome investigations of the optic nerve/tract of CPZ-fed mice have not been reported in the literature. Likewise, no proteomic investigation of optic nerve/tract was found for any other animal models (e.g. EAE (Krishnamoorthy and Wekerle 2009)). Proteomic investigation of the visual pathway has been proposed as an approach to better understand the pathoaetiology of eye diseases and identify early biomarkers (Semba et al. 2013). Accordingly, this study used coupled histological and proteomic analyses to assess alterations in the visual pathway following 12 weeks of 0.1% CPZ-feeding; this treatment paradigm was chosen because it induces comparable demyelination and gliosis in the corpus callosum but less suppression of the peripheral immune system than 0.2% CPZ (Almuslehi et al. 2020; Sen et al. 2019a). Due to this slow demyelination process, we hypothesized that CPZ-induced changes in the proteome of the visual pathway would be consistent with those underlying changes in visual perception in MS and other neurodegenerative eye diseases.

Here, the histological investigation focussed on the visual pathway (Huberman and Niell 2011; Seabrook et al. 2017) from the optic nerve/tract to the visual cortex, including its subcortical relay nuclei (pretectal, superior colliculi and thalamic lateral geniculate nuclei). The proteomic investigation was carried out using optic nerve/tract tissue that extended from the posterior part of eyeballs to the end of the visible part of the optic tract (posterior to the optic chiasm and before the lateral geniculate body) in order to ensure there was no contamination with other CNS tissue/proteins. The other visual components (e.g. pretectal nuclei, superior colliculi, lateral geniculate nuclei and visual cortex) were excluded from the proteomic analysis.

We used a well-established, high-resolution top-down proteomic analysis — two-dimensional gel electrophoresis coupled with liquid chromatography and tandem mass spectrometry (2DE/LC-TMS) — rather than the inferred sequencing of canonical proteins (i.e. shotgun proteomics) in order to resolve and identify proteoforms (i.e. protein species), the active biological entities (Aebersold et al. 2018; Carbonara et al. 2021; Coorssen and Yergey 2015; Oliveira et al. 2014; Sen et al. 2021; Zhan et al. 2019). Following identification, proteome changes in the optic nerve/tract of the CPZ model were compared to published proteome data on eye diseases, MS patients and MS animal models from analyses of a variety of samples including cerebrospinal fluid (CSF), CNS tissue and tears to search for possible correlations.

## Materials and Methods

### Animals and Feeding

Adult (7-week old) male C57Bl/6 mice (*n* = 20) were purchased from the Animal Resources Centre, Australia (www.arc.wa.gov.au). Mice were acclimatized for 1 week and housed (2 animals/ventilated GM500 cage, Tecniplast, Italy) in a controlled environment (12-h light/dark cycle, 50–60% humidity and 21–23 °C) in the animal facility (School of Medicine, Western Sydney University). Standard rodent powder chow (Gordon’s Specialty Stockfeed, Australia) and water were available ad libitum.

Age-/weight-matched mice were randomly divided into control (Ctrl) or CPZ groups (*n* = 10 mice/group). CPZ (Sigma-Aldrich, St. Louis, MO, USA; 0.1% w/w) was mixed with powdered chow and fed to mice for 12 weeks to induce a slow progressive oligodendrocytosis and demyelination (i.e. more reminiscent of MS (Sen et al. 2020a)). The powdered chow was prepared daily without (for Ctrl group) and with CPZ and provided in excess (ad libitum) in a single shared feeder per cage. At the end of 12 weeks, all mice were euthanized for histological and proteomic analyses.

### Histology and Immunohistochemistry

Quantitative histological examination was performed as previously described (Almuslehi et al. 2020; Sen et al. 2019a, 2020b). Briefly, mice (*n* = 5/group) were deeply anaesthetized with isoflurane (2–3%) and perfused transcardially with cold 0.9% saline followed by 4% paraformaldehyde (PFA, in 0.1 M phosphate buffer). Brains (with attached optic tracts) were extracted from the skulls, sectioned coronally (40 µm) and transferred either mounted onto 0.4% gelatine-coated slides (for silver staining) or to 6-well plates containing 0.01 M phosphate buffer saline (PBS) (free-floating for immunohistochemistry).

Silver staining was performed as described previously (Almuslehi et al. 2020; Pistorio et al. 2006; Sen et al. 2019a, 2020b). Briefly, mounted tissue sections (40 µm coronal) were air-dried for 48 h and immersed in 10% natural formalin (Sigma-Aldrich) for 2 weeks at room temperature (RT) (~ 22 °C). Brain sections (with attached optic nerve/tract) from Ctrl and CPZ-fed animals were processed in parallel (i.e. in the same solutions and at the same time) to maintain the consistency of staining in both groups. Slides were then washed with distilled water and incubated in pyridine (VWR, USA): acetic anhydride (Merck, Germany) solution (ratio 2:1) for 30 min at RT. Sections were then rehydrated sequentially with decreasing concentrations of ethanol (80, 60, 40 and 20%) for 20 s in each concentration and washed using distilled water. In the next step, the slides were immersed in ammoniacal silver nitrate (Chem-Supply, Australia) containing developing solution (mixture of sodium carbonate, ammonium nitrate and silver nitrate) for 45 min at RT. Sections were then washed for 30 s with a bleaching agent (potassium ferricyanide; BDH chemicals, UK) to de-stain over developed sections. This was followed by dehydration in sequential increasing concentrations of ethanol (20, 40, 60, 80 and 100%) for 20 s in each. Finally, sections were cleaned by xylene (VWR, USA) for 5 min and covered with mounting medium (Merck, USA), sealed with coverslips (Knittel Glass, Germany) and air-dried for 72 h. Silver-stained sections were imaged with bright field microscopy (Olympus Carl Zeiss, Jena, TH, Germany). ImageJ software (https://imagej.nih.gov/ij/) was used to analyse the images, and the results were plotted as the reciprocal of the light intensity to measure the amount of myelin (Sen et al. 2019a, 2020b).

For immunofluorescence staining, free-floating coronal CNS tissue sections (40 µm) were bathed with warm (40–50 °C) 0.01 M PBS to remove residual gelatine. Non-specific binding of antibodies was minimized by immersing tissue sections into 10% normal goat serum (Sigma, USA) at RT for 2 h. Sections were then transferred into primary antibodies diluted with 0.01 M PBS plus 0.1% Triton X-100 (TX-100, Amresco). Ionized calcium-binding adaptor molecule 1 (rabbit anti-IBA 1, 1:1000; Wako, Chuo-Ku, OSA, Japan) and glial fibrillary acidic protein (mouse anti-GFAP Alexa 488, 1:1000; Merck-Millipore, Burlington, MA, USA) primary antibodies were used for the detection of microglia and astrocytes, respectively. All primary antibodies were incubated for 12 h at RT while shaking on an orbital shaker (50 rpm). Sections were then washed in 0.01 M PBS and incubated in secondary Alexa Fluor 555 conjugated antibody (for IBA 1 only) for 2 h at RT with agitation on the orbital shaker. Following three washes in 0.01 M PBS, sections were mounted on glass slides and covered with 1.5 µg/mL vectashield plus 4′,6-diamidino-2-phenylindole (DAPI, Vector Laboratories) to stain the nuclei and then sealed with coverslips (Knittel Glass, Germany). Tissue sections were dried for 30 min at RT and stored at 4 °C in the dark until imaged. GFAP and IBA 1 stained sections were imaged using a fluorescence microscope (Olympus Carl Zeiss, Germany). Quantification of the fluorescence intensity of the stained sections was carried out using ImageJ software (Sen et al. 2019a, 2020b). The anatomical locations of visual pathway components were determined using the Mouse Brain Atlas and previous analyses of the mouse CNS (Paxinos and Franklin 2012; Sen et al. 2020b).

### Top-Down Proteomics: Two-Dimensional Gel Electrophoresis (2DE) and Liquid Chromatography-Tandem Mass Spectrometry

**Fig. 1:**
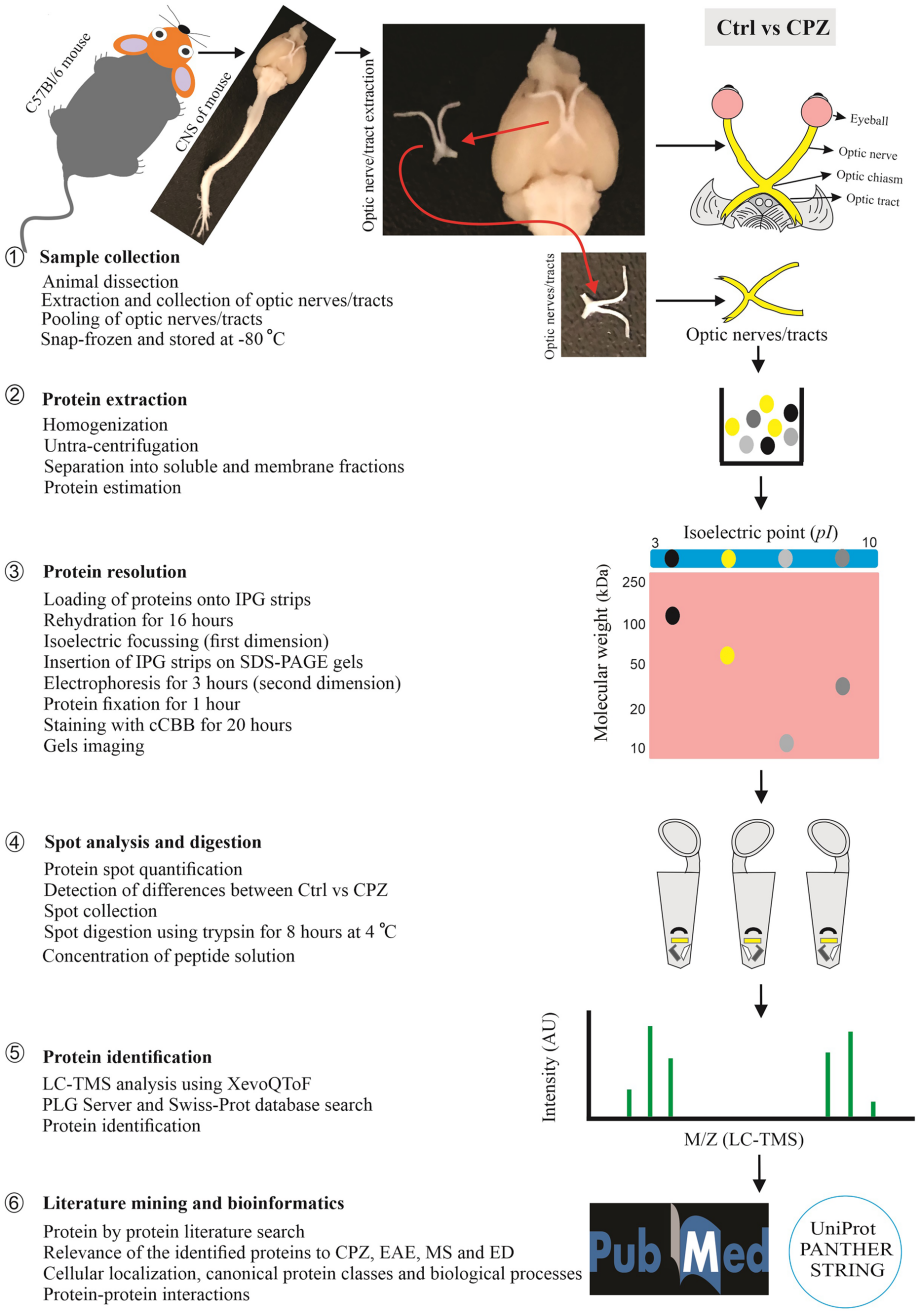
Overview of the top-down proteomic analysis of the optic nerve/tract. The top-down proteomic analysis consisted of 6 general steps. (1) Optic nerve/tract samples were collected after dissection (pooled from 5 animals/treatment group) and snap frozen with liquid nitrogen. (2) Samples were homogenized in a deep-frozen state (i.e. automated frozen disruption), solubilized and fractionated into total soluble and membrane proteomes. (3) Proteoforms were resolved based on charge (*pI*) using immobilized pH gradient strips and then by size (~ MW) using SDS-PAGE and detected using cCBB. (4) Spots were analysed using Delta 2D software to identify quantitative differences between the Ctrl and CPZ groups; these spots were excised from the gel and digested using trypsin. (5) Peptides were analysed by LC-TMS, and the corresponding canonical amino acid sequences of the resolved proteoforms were identified using ProteinLynx Global Server (PLGs) and Swiss-Prot databases. (6) Literature mining and bioinformatic analyses were used to assess potential functions and interactions. Abbreviations: Ctrl, control mice; CPZ, cuprizone-fed mice; MW, molecular weight; *pI*, isoelectric point; SDS-PAGE, sodium dodecyl sulphate poly acrylamide gel; LC-TMS, liquid chromatography-tandem mass spectrometry; EAE, experimental autoimmune encephalomyelitis; ED, eye disease; cCBB, colloidal Coomassie Brilliant Blue dye; IPG strips, immobilized pH gradient strips

High-resolution 2DE was carried out using the optimized method described in detail in earlier studies (Butt and Coorssen 2005, 2013; Gauci et al. 2013; Noaman et al. 2017; Sen et al. 2019a; Wright et al. 2014) and summarized in Fig. 1. Briefly, optic nerves/tracts (from the posterior part of the eyeballs to the contact area of the optic tract with the brain tissue, i.e. before the lateral geniculate body) were harvested and rinsed immediately with ice cold 0.01 M PBS (containing a protease/kinase/phosphatase inhibitor cocktail). The nerves/tracts of each group were pooled due to the very small amount of material and homogenized in the deep-frozen state and solubilized in HEPES hypotonic lysis buffer (isotonicity of the solution restored by adding 2 × PBS and ultracentrifuged at 125,000* g*, 4 °C for 3 h to separate total soluble proteins (SP) from membrane proteins (MP). The resulting pellet was resuspended into 1 × PBS and ultracentrifuged (125,000* g*, overnight at 4 °C) to solubilize and separate the remaining SP proteins (Butt and Coorssen 2005); the two supernatants were then combined to yield a total soluble proteome sample. The EZQ Protein Quantitation Kit (Life Technologies, Eugene, OR, USA) was used according to the manufacturer’s instructions to measure the total protein concentration in each fraction (SP and MP), with bovine serum albumin (Amresco, Solon, OH, USA) as the standard. Proteoforms were then resolved in the first dimension based on net charge (isoelectric point, *pI*) and by size (i.e. approximate molecular weight) in the second dimension. Total protein extract (100 µg) was passively loaded onto immobilized pH gradient (IPG) strips (7 cm, non-linear, pH 3–10; Bio-Rad, Hercules, CA, USA) at RT for 16 h. The strips were then subjected to isoelectric focusing (IEF, first dimension) using the Protean IEF apparatus (Bio-Rad, USA; 17 °C, 10,000 V, 37,500 VH) with multiple replacements of the electrode wicks during voltage ramping to facilitate effective desalting. Then, following incubation of the resolved IPG strips with reducing and alkylating reagents as well as SDS, the strips were loaded horizontally on the top of hand-cast (8.4 cm × 7 cm × 0.1 cm) SDS-PAGE gels, and electrophoresis was carried out at 4 °C with an initial voltage of 150 V for 5 min followed by 90 V for 3 h using the Mini-PROTEAN Cell (Bio-Rad, USA). Resolved proteoforms were then detected by incubation in colloidal Coomassie Brilliant Blue (cCBB, G-250, Amresco, USA) for 20 h followed by de-staining with 0.5 M NaCl (5 times × 15 min). The cCBB dye was used for in-gel proteoform detection due to its established high sensitivity, reproducibility and compatibility with mass spectrometry analysis (Gauci et al. 2013; Sen et al. 2019a; Wright et al. 2014). Gels were imaged individually on the Typhoon™ FLA-9000 gel imager (GE Healthcare, USA) using the same setting for all gels (100 µm resolution, 685/ > 750 nm excitation/emission and 600 V photomultiplier tube). For each group (Ctrl or CPZ), replicate 2DE gels (*n* = 3) for each SP and MP fraction (i.e. 6 gels per group in total) were resolved to ensure reproducibility and thus reliability of the results.

Quantitative analysis of gel images was carried out by using Delta2D (version 4.0.8, DECODON Gmbh, Germany) as described previously (Sen et al. 2019a). Briefly, gel images were grouped for each fraction in each experimental group (e.g. SP gels for Ctrl group). In each comparison (e.g. SP of Ctrl group vs SP of CPZ group), gel images were warped and fused using ‘union fusion’ to create a master gel image. Spots of the master image were then transferred to each image in their group to ensure that the spots consistently matched (100% matching) in all technical replicates (*n* = 3) in each group. Gel edges and the molecular weight marker lanes were excluded manually. Background-subtracted spot volumes were then tabulated as grey values, including fold changes, relative standard deviation (RSD) and *p* values (*t-* test). Following the assessment of changes in fluorescent spot volumes in the CPZ group gels compared to Ctrls, candidate spots were selected for further analysis (i.e. proteolytic digestion and LC-TMS). This selection was based on specific criteria: *p* value (*p* <0.05), ratio of grey value > 1.5-fold (Ctrl vs CPZ) and relative standard deviation (RSD) < 30%. Alterations in the ratio (fold change between CPZ and Ctrl gels) for each selected spot were determined by dividing the average (*n* = 3) grey value of a given spot in the CPZ optic nerve/tract gels by the average grey value of the corresponding spot in the Ctrl gels. Molecular weight (MW) and isoelectric point (*pI*) of all selected spots were determined using standards, as previously described (Sen et al. 2019a). Briefly, three calibration gels (including both MW markers and *pI* standards for 2DE, Bio-Rad) were used to establish the experimental MW and *pI* of resolved proteoforms. The coefficient of variation (standard deviation/mean) of 2DE standards (*n* = 3) was 1.9% for the MW migration and 0.8% for the *pI*. In the experimental gels (*n* = 12), the coefficient of variation was 3.3% for MW marker. Experimental MW and *pI* values were plotted relative to theoretical values (obtained from ProteinLynx Global Server) to identify the variations that indicate post-translational modifications of proteins (i.e. proteoforms). The changes were considered significant when the experimental values fell below or above the confidence intervals (95%) of the MW and *pI* calibration curve.

The selected spots were excised manually and digested using 12.5 ng/µL trypsin (Promega Corporation, USA) at 4 °C for 8 h. The resulting peptides were analysed using LC-TMS on a Xevo QToF mass spectrometer (Waters, USA) as described (Asgarov et al. 2021; Sen et al. 2019a; Wright et al. 2014). ProteinLynx Global Server (PLG) software (version 2.5, Waters Corporation, USA) and the Swiss-Prot (*Mus musculus*, mouse) database (www.wehi.edu.au) were used for data acquisition and processing, respectively, with the following settings: minimum fragments per peptide, 3; minimum peptides per protein, 2; minimum fragments per protein, 7; maximum false-positive rate, 4; maximum protein mass, 250,000; fixed modifications, carbamidomethyl (C); variable modifications, oxidation (M); missed cleavages, 1; peptide tolerance, automatic; and fragment tolerance, automatic. Finally, identified proteoforms in each spot had to satisfy specific criteria (i.e. protein score ≥ 100, sequence coverage ≥ 5%, unique matched peptides ≥ 3 and the false-positive rate < 0.01%) in order to be included for further assessment.

### Bioinformatics and Literature Mining

Functional annotations of identified proteoforms were assessed via bioinformatic analyses (Asgarov et al. 2021; Sen et al. 2019a, 2021). Briefly, the UniProt accession number, gene ID and subcellular localization of the canonical protein corresponding to each identified proteoform were obtained from the publicly accessible UniProt database (www.uniprot.org). Protein analysis through evolutionary relationships (PANTHER, www.pantherdb.org) was used to define canonical protein classes and biological processes (Mi et al. 2018). In addition, identified protein species were further characterized and grouped to investigate their potential interaction with other proteins (protein–protein interactions (PPI)) using the search tool for the retrieval of interacting genes/proteins (STRING, string-db.org) database (Szklarczyk et al. 2018). A PPI map was then generated using STRING in which each node represents one canonical protein (indicated by gene ID) and the connecting lines represent confidence/strength of association (i.e. increasing line thickness reflects the potential for interaction). Finally, a comprehensive PubMed literature search (www.ncbi.nlm.nih.gov/pubmed/) was applied to identify the molecular functions of the proteoforms and their relevance to previous associations with animal models of MS (e.g. CPZ, EAE) and MS itself (Table 1). Moreover, previously identified changes in the abundance of canonical proteins in different CPZ (4), EAE (13) and MS (13) studies using various samples other than optic nerve/tract (e.g. cerebrum, CSF, tears) were also compared with the current data. Out of six MS-like animal models — EAE, CPZ, ethidium bromide, lysolecithin, diphtheria toxin and Theiler’s virus (Procaccini et al. 2015; Ransohoff 2012; Traka et al. 2010) — proteomic studies were only found for two: EAE and CPZ. Additionally, to compare the identified proteoforms in this study with eye diseases (e.g. optic neuritis, dry eye disease and neuromyelitis optica), these studies were also included (Bai et al. 2009; Jung et al. 2017; Olesen et al. 2019).

**Table 1:** Identified optic nerve/tract proteoforms and comparison with published literature

**Spot ID**	**Response to CPZ (ratio/*p*** **value)**	**Gene ID**	**Identified proteoform**	**Protein accession**	**Theoretical MW (kDa)/*pI***	**Observed MW (kDa)**/***pI***	**PLG Score**	**Sequence coverage (%)**	**Matched peptides**	**Molecular function**	**References**			
											**CPZ**	**EAE**	**MS**	**ED**
SP1	(1.57/ 0.049)↓	Nefm	Neurofilament medium polypeptide	P08553	96.0/4.6	117.3/ 4.7	3980	48	56	IV	↓Cerebrum (Szilagyi et al. 2020)	↑Spinal cord (Farias et al. 2012), ↓spinal cord (Hasan et al. 2019)	-	-
		Tubb3	Tubulin beta-3 chain	Q9ERD7	50.9/4.6		2732	37	24	I	-	↓Spinal cord (Hasan et al. 2019), ↓brain stem (Vanheel et al. 2012)	-	-
		Tubb2a	Tubulin beta-2A chain	Q7TMM9	50.3/4.6		2683	46	25	I	-	↑Spinal cord (Jain et al. 2012)	-	-
SP2	(3.58/ 0.002)↓	Tuba1c	Tubulin alpha-1C chain	P68373	50.6/4.8	110.6/ 4.9	2548	40	17	I	-	-	-	-
		Tuba1b	Tubulin alpha-1B chain	P05213	50.8/4.8		2511	35	15	I	-	↑Spinal cord (Farias et al. 2012)	-	-
		Tuba1a	Tubulin alpha-1A chain	P68369	50.8/4.8		2511	35	15	I	-	↑Spinal cord (Farias et al. 2012)	-	-
SP3	(2.25/ 0.05)↓	Serpina3k	Serine protease inhibitor A3K	P07759	47.1/4.8	95.9/ 4.6	2181	29	14	VII	↓Cerebrum (Szilagyi et al. 2020)	-	-	-
		Serpina3c	Serine protease inhibitor A3C	P29621	46.9/8.0		321	6	6	VII	-	-	-	-
		Serpina3n	Serine protease inhibitor A3N	Q91WP6	46.9/5.5		318	5	4	VII	-	↑Stool (Gonzalez et al. 2019)	-	-
SP4	(1.67/ 0.001)↑	Hspa4	Heat shock 70 kDa protein 4	Q61316	94.9/5.0	93.5/ 5.2	2537	45	47	III	-	↓Spinal cord (Hasan et al. 2019), ↑brain stem (Vanheel et al. 2012)	-	-
		Actb	Actin_ cytoplasmic 1	P60710	42.1/5.2		2482	51	26	I	-	↑CSF (Stoop et al. 2012), ↓cerebrum (Hasan et al. 2019)	↑CSF (Dumont et al. 2004; Hammack et al. 2004)	-
		Actg1	Actin_ cytoplasmic 2	P63260	42.1/5.2		2475	47	25	I	-	-	↓CSF (Liu et al. 2009)	-
SP5	(3.12/ 0.001)↓	Hsp90ab1	Heat shock protein HSP 90-beta	P11499	83.6/4.8	90.8/ 5.1	639	26	19	III	↓Spleen (Partridge et al. 2016)	↑Cerebellum, spinal cord (Hasan et al. 2019); ↑spinal cord (Jain et al. 2012)	↑Tear (Salvisberg et al. 2014), ↑PBMC (De Masi et al. 2009)	-
		Hsph1	Heat shock protein 105 kDa	Q61699	97.4/5.2		601	19	18	III	↓Cerebrum (Szilagyi et al. 2020)	-	-	-
		Hsp90aa1	Heat shock protein HSP 90-alpha	P07901	85.2/4.7		555	28	18	III	-	↓Spinal cord (Jain et al. 2012), ↓brain stem (Vanheel et al. 2012)	-	-
SP6	(2.01/ 0.038)↓	Mag	Myelin-associated glycoprotein	P20917	70.1/4.9	90.2/ 4.4	1539	19	12	IV	↓Cerebrum (Werner et al. 2010)	↓Spinal cord (Hasan et al. 2019; Jain et al. 2012)	↓Cerebrum (Ly et al. 2011)	-
		Hba	Haemoglobin subunit alpha	P01942	15.1/8.6		1312	29	4	VI	-	↓Spinal cord (Jain et al. 2012)	↑Urine (Singh et al. 2015)	↓DED, tear (Jung et al. 2017)
SP7	(1.59/ 0.021)↓	Vcp	Transitional endoplasmic reticulum ATPase	Q01853	90.0/4.7	90.1/ 5.2	2829	53	71	III	-	↑Spinal cord (Jain et al. 2012)	-	↑DED, tear (Jung et al. 2017)
		Actn4	Alpha-actinin-4	P57780	10.5/5.1		2240	50	56	I	-	↑Spinal cord (Hasan et al. 2019)	-	-
		Actn1	Alpha-actinin-1	Q7TPR4	10.4/5.7		1011	35	35	I	-	↑Spinal cord (Hasan et al. 2019)	-	-
SP8	(1.67/ 0.041)↑	Aco2	Aconitate hydratase	Q99KI0	86.2/7.8	87.7/ 6.8	2217	33	29	II	↑Cerebrum (Sen et al. 2019a)	↑Brain stem (Vanheel et al. 2012)	↑PBMC (Berge et al. 2019)	-
SP9	(1.72/ 0.035)↑	Pfkm	ATP-dependent 6-phosphofructokinase	P47857	86.1/7.9	85.5/ 6.9	561	26	23	II	-	-	-	-
		Aco2	Aconitate hydratase	Q99KI0	86.2/7.8		496	17	13	II	↑Cerebrum (Sen et al. 2019a)	↑Brain stem (Vanheel et al. 2012)	↑PBMC (Berge et al. 2019)	-
SP10	(1.69/ 0.037)↑	Syn1	Synapsin-1	O88935	74.2/10.4	77.2/ 8.5	930	27	13	XI	-	↑Serum (Raphael et al. 2017), ↓spinal cord (Hasan et al. 2019; Jain et al. 2012)	-	-
		Map6	Microtubule-associated protein 6	Q7TSJ2	96.7/6.3		247	14	8	I	-	↓Spinal cord (Hasan et al. 2019; Jain et al. 2012)	-	-
SP11	(1.69/ 0.015)↑	Chmp4b	Charged multivesicular body protein 4b	Q9D8B3	24.9/4.6	58.4/ 4.7	2045	42	14	VIII	↓Cerebrum (Sen et al. 2019a)	-	-	-
		Otub1	Ubiquitin thioesterase OTUB1	Q7TQI3	31.5/4.7		1510	29	8	III	-	-	-	-
		Anxa5	Annexin A5	P48036	35.8/4.6		1303	25	11	IX	↑Cerebrum (Werner et al. 2010), ↓Cerebrum (Szilagyi et al. 2020)	↑Spinal cord (Fazeli et al. 2013; Jain et al. 2012; Linker et al. 2009)	-	-
SP12	(2.29/ 0.015)↑	Sncb	Beta-synuclein	Q91ZZ3	14.1/4.2	27.0/ 4.6	3031	34	6	III	-	↓Spinal cord (Hasan et al. 2019; Jain et al. 2012)	-	-
		Snca	Alpha-synuclein	O55042	14.5/4.5		1063	16	4	III	-	-	-	-
SP13	(1.61/ 0.04)↑	Pvalb	Parvalbumin alpha	P32848	11.9/4.8	17.6/ 4.9	9188	77	12	V	-	↑Cerebrum (Hasan et al. 2019), ↑spinal cord (Fazeli et al. 2013; Hasan et al. 2019)	-	-
		Txn	Thioredoxin	P10639	12.0/4.6		3687	36	6	V	-	↑Spinal cord (Jain et al. 2012), ↓spinal cord (Hasan et al. 2019)	↑PBMC (De Masi et al. 2009)	-
		Sh3bgrl	SH3 domain-binding glutamic acid-rich-like protein	Q9JJU8	12.9/4.7		1747	50	5	V	-	↑Spinal cord (Hasan et al. 2019)	-	-
MP1	(2.75/ 0.01)↑	Nefm	Neurofilament medium polypeptide	P08553	96.0/4.6	132.3/ 4.7	2693	40	34	IV	↓Cerebrum (Szilagyi et al. 2020)	↑Spinal cord (Farias et al. 2012), ↓spinal cord (Hasan et al. 2019)	-	-
		Vim	Vimentin	P20152	53.7/4.9		2063	55	31	I	↑Cerebrum (Szilagyi et al. 2020; Werner et al. 2010)	↑Cerebrum (Hasan et al. 2019), ↑spinal cord (Fazeli et al. 2010; Jain et al. 2012; Liu et al. 2007)	↑CSF (Noben et al. 2006)	-
		Tuba3a	Tubulin alpha-3 chain	P05214	50.64/4.8		646	15	6	I	-	↓Spinal cord (Jain et al. 2012)	-	-
MP2	(3.55/ 0.01)↑	Ina	Alpha-internexin	P46660	55.6/5.2	97.8/ 4.6	148	5	4	I	-	↑Spinal cord (Farias et al. 2012; Linker et al. 2009), ↓spinal cord (Hasan et al. 2019; Jain et al. 2012)	-	-
MP3	(1.63/ 0.03)↑	Aco2	Aconitate hydratase	Q99KI0	86.2/7.8	90.1/ 6.9	1278	26	22	II	↑Cerebrum (Sen et al. 2019a)	↑Brain stem (Vanheel et al. 2012)	↑PBMC (Berge et al. 2019)	-
MP4	(2.19/ 0.03)↑	Tf	Serotransferrin	Q921I1	78.9/6.9	82.8/ 6.8	539	13	12	VI	-	↑Spinal cord (Hasan et al. 2019; Linker et al. 2009; Liu et al. 2009), ↓PBMC (Dagley et al. 2014), ↓brain stem (Vanheel et al. 2012)	↑CSF (Kroksveen et al. 2013; Liu et al. 2009; Noben et al. 2006), ↓CSF (Kroksveen et al. 2012)	-
MP5	(1.84/ 0.02)↑	Atp5f1a	ATP synthase subunit alpha	Q03265	59.9/9.6	66.3/ 6.8	3951	53	39	II	↑Cerebrum (Sen et al. 2019a)	↑Spinal cord (Jain et al. 2012)	-	-
		Cap1	Adenylyl cyclase-associated protein 1	P40124	51.9/7.3		832	31	11	I	-	-	↑PBMC (De Masi et al. 2009)	-
		Anxa11	Annexin A11	P97384	54.4/7.6		379	25	13	IX	-	-	-	-
MP6	(1.71/ 0.02)↑	Aldh6a1	Methylmalonate-semialdehyde dehydrogenase	Q9EQ20	58.4/8.1	64.1/ 6.7	571	30	16	II	-	-	-	-
		Cct4	T-complex protein 1 subunit delta	P80315	58.6/8.0		200	19	12	I	-	-	-	-
		Gpi	Glucose-6-phosphate isomerase	P06745	63.0/8.2		188	20	11	II	-	↓Spinal cord (Jain et al. 2012)	-	-
MP7	(1.51/ 0.01)↑	Gfap	Glial fibrillary acidic protein	P03995	50.0/5.1	57.7/ 4.8	25,975	87	84	I	↑Cerebrum (Sen et al. 2019a; Szilagyi et al. 2020; Werner et al. 2010)	↑Brain stem (Vanheel et al. 2012), ↑spinal cord (Farias et al. 2012; Fazeli et al. 2013; Hasan et al. 2019; Jain et al. 2012; Linker et al. 2009)	-	-
		Nefl	Neurofilament light polypeptide	P08551	61.6/4.4		14,009	59	45	IV	↓Cerebrum (Sen et al. 2019a)	↑ Spinal cord (Farias et al. 2012), ↓spinal cord (Hasan et al. 2019)	↑CSF (Jia et al. 2012; Liu et al. 2009)	↑ON, CSF (Olesen et al. 2019), ↑NMO, CSF (Bai et al. 2009)
		Pdia6	Protein disulphide isomerase A6	Q922R8	48.5/4.8		9115	44	22	II	-	-	↓PBMC (Berge et al. 2019)	-
MP8	(1.56/ 0.002)↑	Tuba1b	Tubulin alpha-1B chain	P05213	50.8/4.8	55.9/ 4.9	3205	33	12	I	-	↑Spinal cord (Farias et al. 2012)	-	-
		Tuba1a	Tubulin alpha-1A chain	P68369	50.8/4.8		3205	33	12	I	-	↑Spinal cord (Farias et al. 2012), ↓brain stem (Vanheel et al. 2012)	-	-
		Tuba1c	Tubulin alpha-1C chain	P68373	50.6/4.8		3205	38	13	I	-	-	-	-
MP9	(1.66/ 0.004)↑	Des	Desmin	P31001	53.6/5.0	53.0/ 4.7	791	13	8	I	-	-	-	-
		Prph	Peripherin	P15331	54.4/5.3		212	11	10	I	-	↓Brain stem (Hasan et al. 2019), ↓spinal cord (Jain et al. 2012)	-	-
		Sparc	Secreted protein acidic and cysteine rich	P07214	35.3/4.6		131	8	3	VIII	-	-	-	-
MP10	(1.52/ 0.001)↑	Nsfl1c	NSFL1 cofactor p47	Q9CZ44	40.7/4.9	51.3/ 4.8	3878	51	20	XI	-	↑Spinal cord (Jain et al. 2012)	-	-
		Actg1	Actin_ cytoplasmic 2	P63260	42.1/5.2		2178	44	23	I	-	-	↓CSF (Liu et al. 2009)	-
		Actb	Actin_ cytoplasmic 1	P60710	42.1/5.2		2178	44	23	I	-	↑CSF (Stoop et al. 2012)	↑CSF (Dumont et al. 2004; Hammack et al. 2004)	-
MP11	(1.86/ 0.007)↑	Idh2	Isocitrate dehydrogenase	P54071	51.7/9.0	50.6/ 7.0	1103	21	9	II	-	↑Spinal cord (Jastorff et al. 2009)	-	-
		Uqcrc2	Cytochrome b-c1 complex subunit 2	Q9DB77	48.3/9.7		753	18	10	II	-	↓Spinal cord (Jain et al. 2012)	-	-
		Serpinh1	Heat shock protein 47	P19324	46.6/9.3		413	7	4	III	-	-	-	-
MP12	(1.64/ 0.02)↑	Cnp	2′_3′-cyclic-nucleotide 3′-phosphodiesterase	P16330	47.5/9.4	49.1/ 6.6	4642	38	25	II	↓Cerebrum (Szilagyi et al. 2020)	-	-	-
		Paics	Multifunctional protein ADE2	Q9DCL9	47.7/7.0		1028	22	14	II	-	-	-	-
		Psmc5	26S proteasome regulatory subunit 8	P62196	45.8/7.4		587	27	12	III	-	-	-	-
MP13	(2.19/ 0.001)↑	Cs	Citrate synthase	Q9CZU6	52.0/8.8	48.5/ 6.6	913	19	9	II	-	↓Spinal cord (Hasan et al. 2019)	-	-
		Ckmt1	Creatine kinase U-type	P30275	47.4/8.2		621	23	11	II	↑Cerebrum (Sen et al. 2019a)	↓Spinal cord (Jain et al. 2012)	-	-
		Pgk1	Phosphoglycerate kinase 1	P09411	44.9/7.9		317	31	12	II	-	↑Spinal cord (Farias et al. 2012), ↓spinal cord (Jain et al. 2012)	↓PBMC (De Masi et al. 2009)	-
MP14	(2.07/ 0.02)↑	Pgk2	Phosphoglycerate kinase 2	P09041	45.3/6.4	48.1/ 6.8	451	14	6	II	-	-	-	-
		Ckmt2	Creatine kinase S-type	Q6P8J7	47.9/8.8		193	7	6	II	-	-	-	-
MP15	(1.68/ 0.001)↑	Impact	Protein IMPACT	O55091	36.7/4.8	46.3/ 4.8	268	18	6	I	-	-	-	-
		Rp2	Protein XRP2	Q9EPK2	40.3/4.9		233	17	6	II	-	-	-	-
		Dpysl2	Dihydropyrimidinase-related protein 2	O08553	62.7/6.0		232	18	9	II	-	↓Brain stem (Vanheel et al. 2012), ↓spinal cord (Jain et al. 2012), ↑spinal cord (Farias et al. 2012)	-	-
MP16	(1.83/ 0.005)↑	Fbxo2	F-box only protein 2	Q80UW2	34.0/4.0	43.9/ 4.0	1071	19	5	III	-	-	-	-
MP17	(6.07/ 0.03)↑	Tpm2	Tropomyosin beta chain	P58774	32.9/4.5	41.5/ 4.4	3200	49	20	I	-	-	-	-
		Tpm1	Tropomyosin alpha-1 chain	P58771	32.7/4.5		1823	32	15	I	↑Cerebrum (Szilagyi et al. 2020)	↓Spinal cord (Farias et al. 2012)	-	-
		Tpm3	Tropomyosin alpha-3 chain	P21107	33.1/4.5		624	12	6	I	-	↑Spinal cord (Hasan et al. 2019; Jain et al. 2012)	-	-
MP18	(1.66/ 0.001)↑	Clta	Clathrin light chain A	O08585	25.7/4.3	40.9/ 4.6	397	14	6	X	-	↓Spinal cord (Hasan et al. 2019; Jain et al. 2012)	-	-
MP19	(2.21/ 0.007)↑	Cyb5a	Cytochrome b5	P56395	15.2/4.8	21.5/ 4.9	1444	46	5	II	-	↑Spinal cord (Hasan et al. 2019), ↓spinal cord (Jain et al. 2012)	-	-
		Mylpf	Myosin regulatory light chain 2	P97457	19.1/4.6		552	28	5	I	-	-	-	-
		Vsnl1	Visinin-like protein 1	P62761	22.3/4.8		422	33	8	V	-	-	-	-
MP20	(1.56/ 0.007)↑	Cox5b	Cytochrome c oxidase subunit 5B	P19536	14.1/8.5	17.5/ 5.9	1660	21	4	II	-	↓Spinal cord (Jain et al. 2012)	↓Cerebrum (Broadwater et al. 2011)	-
		Fabp5	Fatty acid-binding protein 5	Q05816	15.5/6.2		997	23	4	XII	-	↓Spinal cord (Fazeli et al. 2013)	↑Tears (Salvisberg et al. 2014), ↓PBMC (Berge et al. 2019)	-
MP21	(1.65/ 0.01)↑	Dynll1	Dynein light chain 1	P63168	10.5/7.2	11.2/ 6.7	3752	15	3	I	-	-	-	-
		Hba	Haemoglobin subunit alpha	P01942	15.1/8.6		2480	56	8	VI	-	↓Spinal cord (Jain et al. 2012)	↑Urine (Singh et al. 2015)	↓DED, Tear (Jung et al. 2017)
		H4c1	Histone H4	P62806	11.4/11.9		1242	29	3	I	-	-	-	-

Some studies in the literature identified proteins without describing the magnitude of their change (e.g. fold increase or decrease) compared to controls; these are indicated by a ↑ sign. Also, some proteins are described as absent in these studies, and therefore, a ↓ sign is used in this table to maintain the consistency with those previous studies. Some spots contained more than one clearly identifiable proteoform; presented here are the top three hits with the highest score, coverage and peptide count. Additional hits are provided in Supplementary Table 1. UniProt and Gene IDs were derived from the UniProt database. PLG score, sequence coverage, theoretical (MW/*pI*) and the number of unique peptides were acquired from the PLG. Observed/experimental MW and *pI* were determined relative to parallel standards resolved in the 2DE gels. Molecular function: I, structural; II, metabolic; III, molecular chaperone; IV, myelin component; V, signalling; VI, iron binding; VII, protease inhibitor; VIII, apoptosis; IX, immune response; X, endocytosis; XI; exocytosis; XII, transportation. References were identified using PubMed searches on CPZ, EAE, ED and MS and used to correlate proteoforms identified here with reports in the existing literature

**Key**: *SP* soluble protein, *MP *membrane protein, *MW* molecular weight, *pI* isoelectric point, *-* not found or investigated, *CSF* cerebrospinal fluid, *PBMC* peripheral blood mononuclear cell, *CPZ* cuprizone, *EAE* experimental autoimmune encephalomyelitis, *MS* multiple sclerosis, *ED* eye disease, *ON* optic neuritis, *DED* dry eye disease, *NMO* neuromyelitis optica

*%* percentage; *↑*, increase; *↓*, decrease

### Statistical Analysis and Graphing

Histology and immunohistochemistry data are presented as mean ± standard error of the mean. Statistical analyses (two-tailed *t* test) and graphing of the histological data were performed using GraphPad Prism (version 8; www.graphpad.com, San Diego, CA, USA) software. For proteomic analysis, grey values (i.e. fluorescence intensity) of gel spots were analysed using the unpaired *t* test (within Delta 2D software). Significant differences between CPZ and Ctrl groups were considered when *p* < 0.05. Image processing software CorelDRAW (version 2019; www.coreldraw.com, Canada) was used for image assembly in each figure.

## Results

### Histological Changes Following CPZ-Feeding

**Fig. 2:**
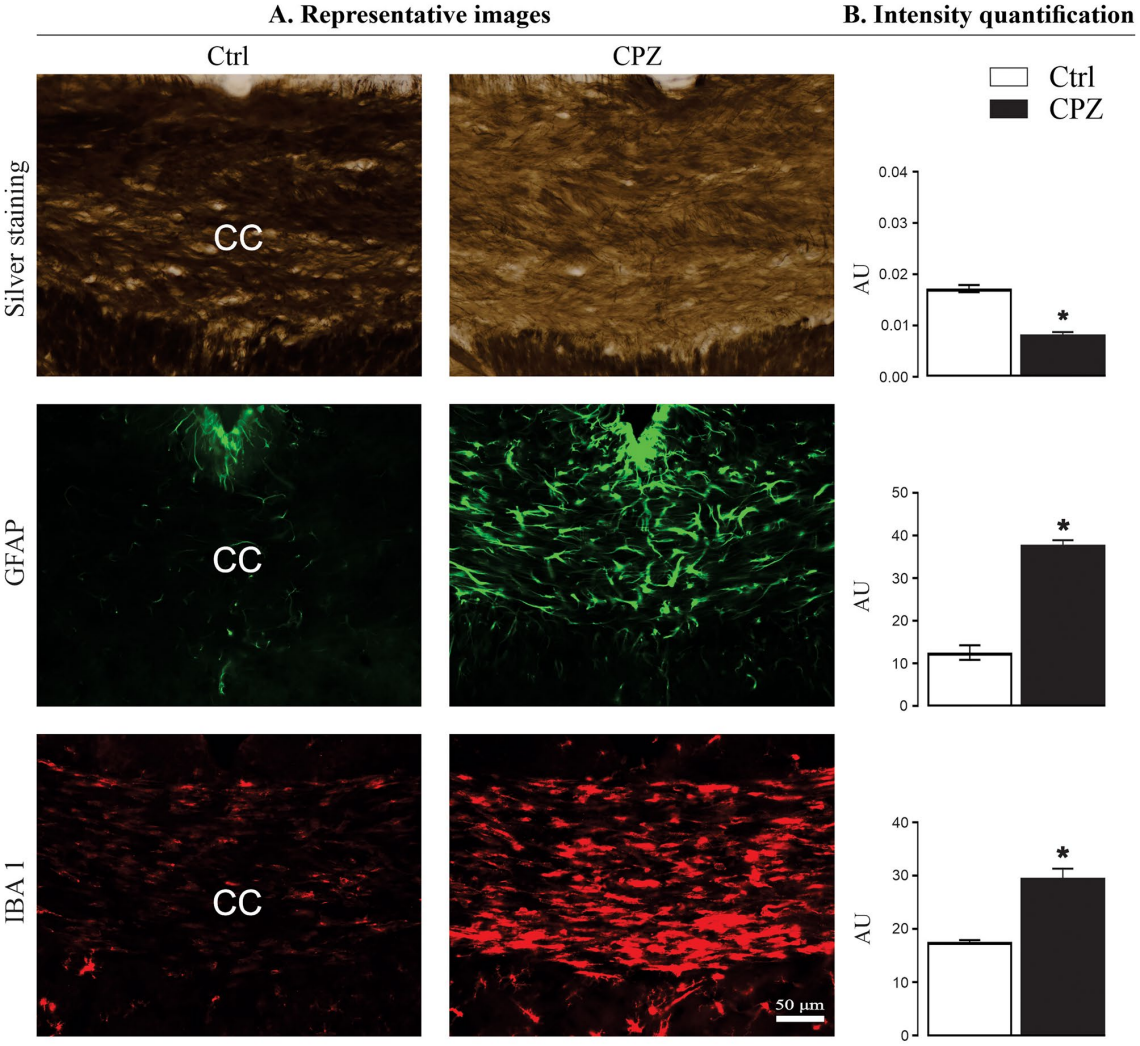
Quantification of demyelination and gliosis. **A** Representative images of coronal brain sections show the midline corpus callosum (CC) stained with silver, GFAP and IBA 1, respectively, from Ctrl and CPZ-fed mice. **B** Quantification of staining intensity (measured in arbitrary units, AU); CPZ-feeding produced significant reduction of silver staining (*n* = 5 sections/animal, 5 animals/group) in the CC. GFAP (3–5 sections/animal, 3–5 animals/group) and IBA 1-stained glia (3–5 sections/animal, 3–5 animals/group) were significantly increased in the CC. Data are presented as mean ± SEM. A two-tailed *t* test was used to determine differences between groups (*p < 0.05). Images and quantitative analysis of different regions of brain sections, stained with silver, are shown in Supplementary Fig. 1, and representative images and quantifications of GFAP and IBA 1 are shown in Supplementary Fig. 2

Myelin silver staining was used to characterize demyelination in the corpus callosum and components of the visual pathway. The midline corpus callosum was used as a positive control as demyelination in this area is a hallmark of CPZ-feeding (Almuslehi et al. 2020; Sen et al. 2019a). In the Ctrl mice, the corpus callosum was darkly stained indicating normal (intact) myelin sheaths, whereas in CPZ-fed mice, the corpus callosum showed markedly reduced silver staining (*p* < 0.0001) indicating demyelination (Fig. 2). Quantitative analysis of the visual pathway revealed no significant demyelination (optic tract, *p* < 0.68; pretectal nucleus, *p* < 0.61; lateral geniculate nucleus, *p* < 0.93; and superior colliculus, *p* < 0.86) although the effect in the visual cortex was on the cusp of significance (*p* < 0.051) (Supplementary Fig. 1). Quantitative immunohistochemical analysis of GFAP-positive astrocytes (*p* < 0.0002) and IBA 1-positive microglia (*p* < 0.0022) in the CPZ-fed mice revealed a significant glial activation in the corpus callosum relative to Ctrl mice (Fig. 2). No significant changes in GFAP (visual cortex, *p* < 0.19; optic tract, *p* < 0.57; pretectal nucleus, *p* < 0.89; lateral geniculate nucleus, *p* < 0.82; and superior colliculus, *p* < 0.13) and IBA 1 (visual cortex, *p* < 0.14; optic tract, *p* < 0.96; pretectal nucleus, *p* < 0.51; lateral geniculate nucleus, *p* < 0.75; and superior colliculus, *p* < 0.22) were observed (Supplementary Fig. 2).

### Changes in the Optic Nerve/Tract Proteome

**Fig. 3:**
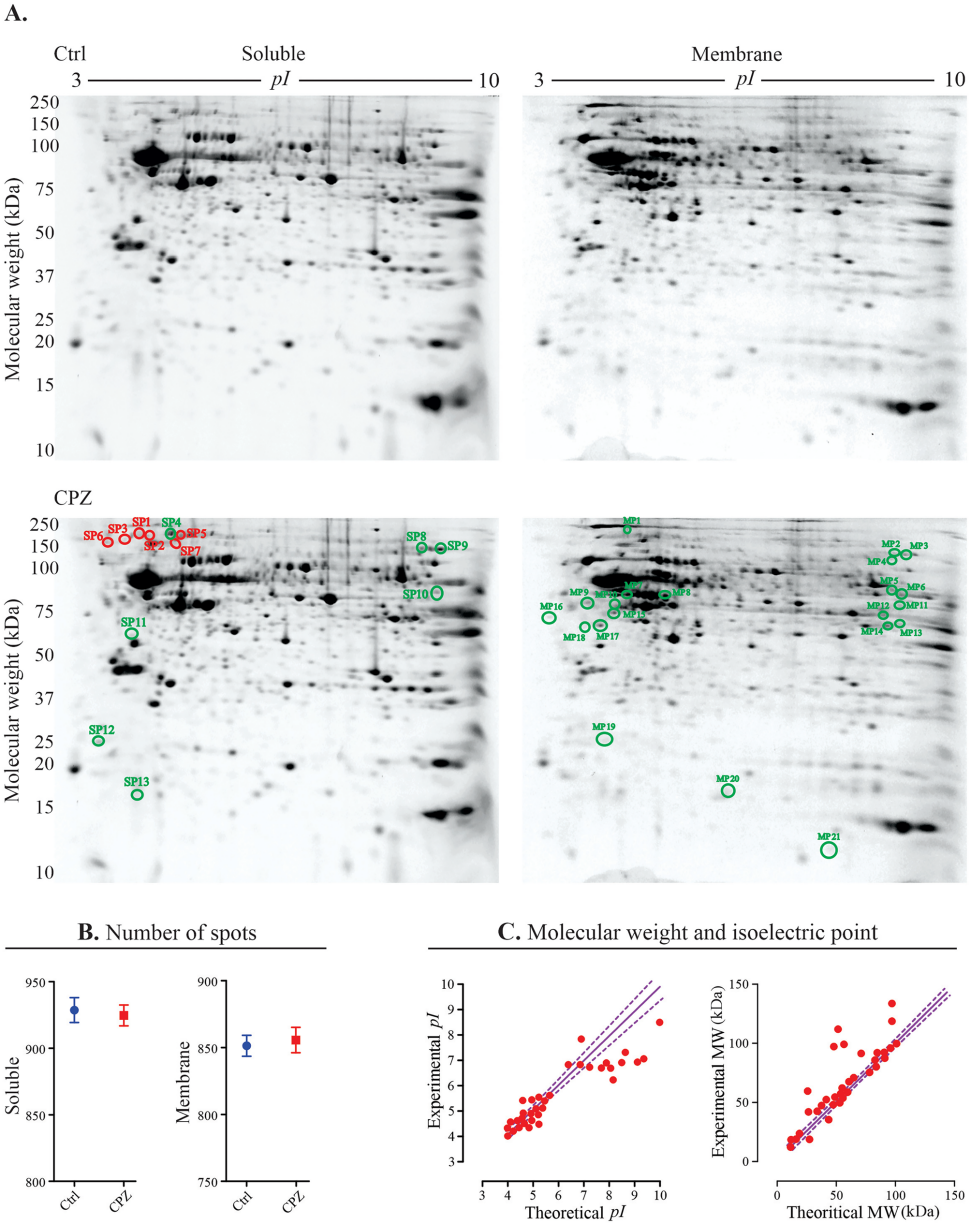
Top-down proteomic analysis. **A** Representative examples of two-dimensional gel images of the soluble and membrane sub-proteomes from the optic nerves/tracts of naïve Ctrl and 0.1% CPZ-fed mice. Proteoforms were resolved based on their isoelectric point (*pI*) and molecular weight (MW). Image analysis revealed a total of 34 spots that increased (green circles) or decreased (red circles) in abundance in the CPZ samples relative to the controls. The identities of the protein species for the circled spots are shown in Table 1 and Supplementary Table 1. **B** There was no significant difference in the total number of spots between Ctrl and CPZ for both the resolved soluble and membrane sub-proteomes. Data are presented as mean ± SEM (*n* = 3 gels/fraction, *n* = 5 animals/group). **C** Comparison between experimental and theoretical *pI* (left graph) and MW (right graph) of the identified proteoforms. The data in these graphs represent the experimental and theoretical MW and *pI* of the first hit of each spot (total number is 34 hits, represented by 34 plots in each graph). Purple dashed line (–-) indicates 95% confidence intervals

A quantitative, high-resolution top-down proteomic analysis was used to assess the optic nerve proteome profile following CPZ-feeding. Optic nerve samples from both Ctrl and CPZ groups produced well-resolved 2DE gels with spots distributed across the full range of *pI* and MW (Fig. 3A). Figure 3A also shows spots (circled green or red) that changed significantly relative to Ctrl in the soluble (SP) and membrane (MP) sub-proteomes (34 spots in total). Comparative image analysis (Ctrl vs CPZ) revealed that 21 MP spots increased in fluorescent volume (i.e. proteoform abundance), whereas in the SP fraction, 7 spots increased and 6 decreased in abundance (*p* < 0.05) (Table 1). The average total spot numbers resolved in the MP fractions from Ctrl and CPZ groups were 851 ± 9 and 856 ± 9, respectively (*n* = 3 gels/fraction); the counts for the SP fractions were 929 ± 9 and 925 ± 8 in the Ctrl and CPZ groups, respectively (Fig. 3B). Figure 3C illustrates the differences between theoretical and experimental MW (upper panel) and *pI* (lower panel) of the identified proteoforms. While several proteoforms (~ 24%) had experimental values for *pI* and MW comparable to the canonical and theoretical values, the rest varied to differing extents from the identity line, indicating the identification of proteoforms, likely with critical post-translational modifications (e.g. glycosylation, ubiquitination). Collectively, following CPZ-feeding, 79.4% of spots showed increased abundance (in both SP and MP fractions), whereas 20.6% decreased in abundance (Supplementary Fig. 3). These 34 spots were digested using trypsin, peptides were analysed using LC-TMS, and high-quality hits were obtained (see Table 1).

2DE spots that were 100% reproducible and had significant change in fluorescent volume across technical (*n* = 3) replicates are summarized in Table 1. The top proteoforms from each spot (i.e. first three proteoforms in the identification list bearing the highest scores) were tabulated (Table 1) along with corresponding literature mining and bioinformatic information (see Methods). The remaining identified proteoforms (i.e. beyond the top three) that satisfied the selection criteria are provided in Supplementary Table 1. Of all the resolved spots that changed significantly (*p* < 0.05) in abundance, 70% changed by 1.5–twofold, 18% by 2.1–threefold and 12% by over threefold. All identified proteoforms had a PLG score exceeding 100, with 43% between 100 and 999, 18% between 1000 and 1999, 21% between 2000 and 2999 and 18% exceeding 3000. Similarly, sequence coverage was always ≥ 5% with 5–9% for 6 proteins only, 10–29% for 40 proteins, 30–49% for 27 proteins and > 50% for 11 proteins. Moreover, each identification was based on at least three matched peptides: 38% based on 3–9 peptides, 35% based on 10–19 peptides, 15% based on 20–30 peptides, 7% based on 30–50 peptides and 5% based on over 50 peptides. The combination of high fold change, PLG scores, the greater number of peptides and high sequence coverage indicated high confidence in the spot selection and proteoform identification.

### Literature Mining and Bioinformatic Analysis

**Fig. 4:**
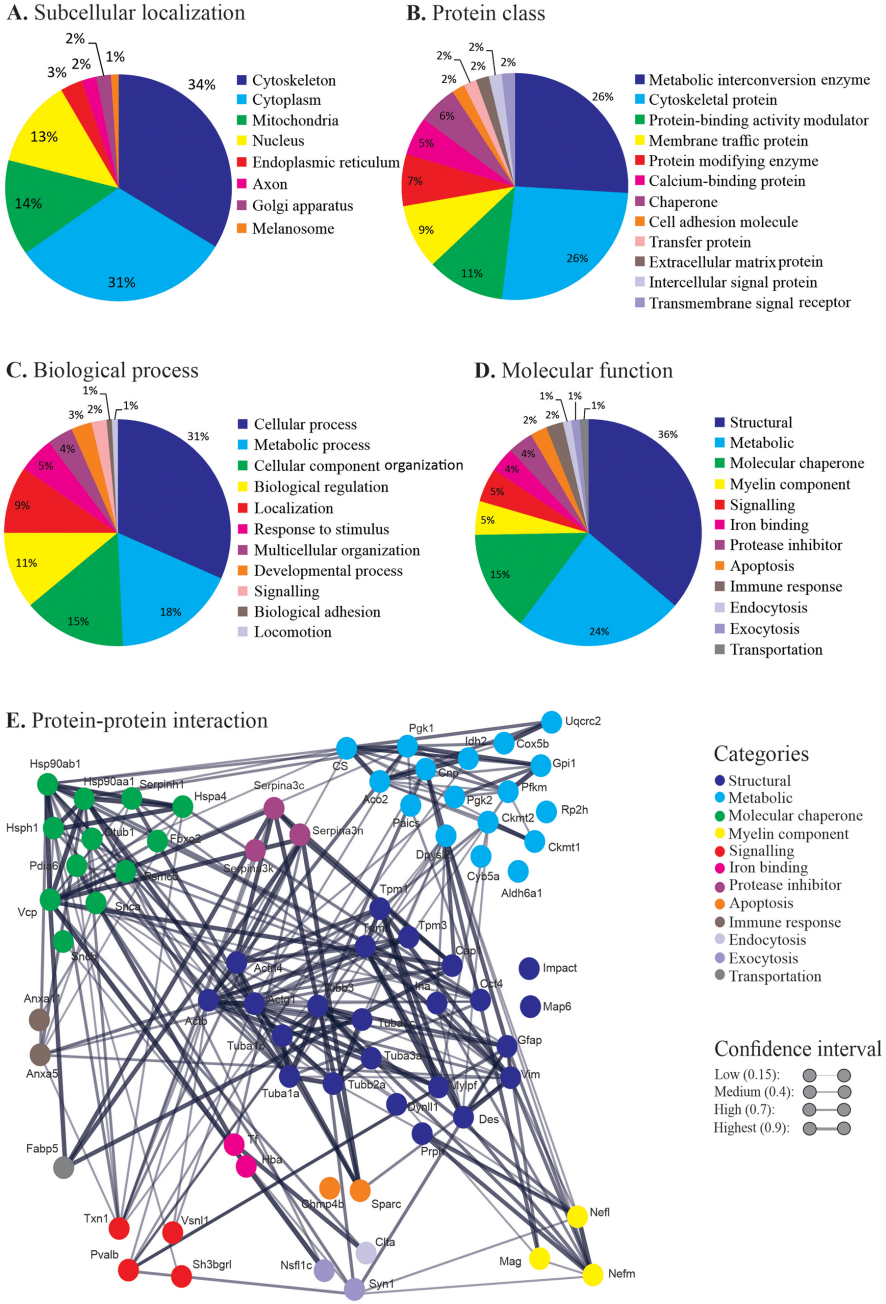
Functional clustering and protein–protein interactions. Pie charts show the distribution of identified proteoforms according to **A** subcellular localizations (characterized using UniProt), **B** protein classes (categorized using PANTHER), **C** biological processes (categorized using PANTHER) and **D** molecular functions (via literature search). **E** Protein–protein interaction association network maps. The strength of interactions is indicated by the thickness of the lines. The colour coding of proteins in the STRING is based on the molecular functions (**D**). Functional clustering and protein–protein interactions of the identified optic nerve proteoforms in the Supplementary Table 1 are shown in Supplementary Fig. 4

A comprehensive literature search was carried out to investigate the linkage of identified proteoforms with MS and its animal models (e.g. EAE and CPZ) (Table 1 and Fig. 4). Twenty-two percent of the proteoforms identified in this study were previously identified as canonical proteins in other CPZ studies, whereas the remaining 78% were newly identified (i.e. not reported previously in CPZ studies (Table 1). Similarly, 24% of the identified proteoforms were previously reported as canonical proteins in MS studies, whereas the remaining 76% were newly identified relative to the previous MS literature; in ED, the previously vs newly identified proteoforms were 5 and 95%, respectively. In EAE, 63% of proteoforms identified here were previously reported as canonical proteins, whereas 37% were newly identified in this study (Table 1). It is thus however critical to emphasize the identification of specific proteoforms using the top-down analytical approach rather than only reporting canonical amino acid sequences that represent any myriad of possible proteoforms. Subcellular localization analysis using UniProt revealed that 34% and 31% of the identified proteoforms are identified as cytoplasmic and cytoskeletal, respectively; 14% and 13% of the proteoforms are, respectively, mitochondrial and nuclear; and a small fraction (1–3%) are found in endoplasmic reticulum, axon, Golgi apparatus and melanosomes (Fig. 4A). Protein class analysis using PANTHER revealed that 26% of proteoforms are involved in metabolic and/or cytoskeletal processes (Fig. 4B). In addition, other proteoforms are implicated in different molecular functions including protein-binding activity modulators (11%), membrane trafficking (9%), protein modifying enzymes (7%), calcium-binding (5%) and chaperones (6%). A small percentage (2%) of proteoforms functions in cell adhesion, transportation, extracellular matrix, intercellular signalling and transmembrane signalling. Furthermore, biological process analysis using PANTHER showed that the majority of the proteoforms were involved in cellular (31%), metabolic (18%) and organization/regulatory (26%) processes (Fig. 4C). Moreover, the literature search indicated various molecular functions of the identified proteoforms including structural (36%), metabolic (24%), chaperone (15%) and signalling or myelin components (5% each) (Fig. 4D). Protein–protein interaction using STRING revealed a central role of structural proteoforms having strong interactions with metabolic, chaperone, signalling and myelin proteoforms, among others (Fig. 4E). The STRING analysis showed that there are direct connections between ~ 90% of proteoforms with the exception of seven (microtubule-associated protein 6, protein IMPACT, cytochrome b5, methylmalonate-semialdehyde dehydrogenase, protein XRP2, haemoglobin subunit alpha and charged multivesicular body protein 4b) that are not linked in any obvious direct manner. The major identified interactions were between the structural proteoforms; 21 of the 23 identified structural proteoforms have well-established interactions. Interestingly, three different clusters of interactions were observed among actin, tubulin and tropomyosin; notably, some of these cytoskeletal proteoforms appear to leave the SP fraction and move to the MP sub-proteome due to the CPZ treatment. In addition, an association of the identified chaperone proteoforms, including heat shock protein family, ubiquitin thioesterase and synuclein, was also indicated by the STRING analysis. Metabolic and mitochondrial regulating proteoforms constituted another network with aconitate hydratase, citrate synthase, phosphoglycerate kinase and creatine kinase. In addition to these multiple functions and interactions of proteoforms (Fig. 4), bioinformatic analyses of proteoforms presented in Supplementary Table 1 were performed to investigate the localization, classes, functions and interactions. Similar to the findings shown in Fig. 4, over 30% of proteoforms are cytoplasmic (Supplementary Fig. 4A), ~ 30% are metabolic interconversion enzymes (Supplementary Fig. 4B), over 30% are involved in cellular processes (Supplementary Fig. 4C), and 30% of the proteoforms have roles in metabolism (Supplementary Fig. 4D). Moreover, STRING bioinformatic analysis (Supplementary Fig. 4E) revealed the complex interaction of metabolic and structural proteoforms with other proteoforms consistent with the interactions as seen in Fig. 4E.

## Discussion

This study used 0.1% CPZ-feeding for 12 weeks in young adult C57Bl/6 mice to investigate whether a slow and progressive degenerative process can induce histological and proteomic alterations in the axonal tracts and nuclear components of the visual pathway. Although histological changes such as demyelination and glial activation were evident in the corpus callosum, none of these changes was observed in the components of visual pathway. However, multiple alterations in the optic nerve/tract proteome were detected using a sensitive, well-established, quantitative top-down proteomic analysis. Subsequent comprehensive literature and bioinformatic analyses revealed that many of the proteoforms identified in the soluble and membrane sub-proteomes of the optic nerve/tract are involved in structural and metabolic functions. Likely aggregation/oligomerization of some proteoforms was also detected.

### Limited Histological Changes

Demyelination and inflammation in the components of the visual pathway (e.g. visual cortex, pretectal nucleus, lateral geniculate nucleus, superior colliculus and optic tract) are associated with optic neuritis (Martínez-Lapiscina et al. 2014; Sapienza et al. 2016), and these conditions have been investigated in animal studies (Guido 2018; Huberman and Niell 2011; Seabrook et al. 2017). In a previous study, we found significant demyelination and gliosis in the visual cortex when mice were fed with 0.2% CPZ for 5 weeks but not in the optic tract (Sen et al. 2020b). In the current study, the lack of detectable demyelination in the visual cortex might be attributable to the staining method or to the concentration of CPZ used. For example, Nile Red staining detected changes in the myelin components of the corpus callosum following only 2 days of CPZ-feeding, whereas other traditional histological staining methods (e.g. silver or luxol-fast blue) detected quantifiable demyelination only after 4–5 weeks (Hiremath et al. 1998; Sen et al. 2020b; Teo et al. 2021). Additionally, demyelination has been detected in the optic nerve after 12 weeks of CPZ-feeding, but no other part of the visual pathway was assessed in that study (Namekata et al. 2014). In the current study, 0.1% CPZ-feeding was extended to 12 weeks to analyse demyelination and gliosis in optic tract, visual cortex, pretectal nucleus, lateral geniculate nucleus and superior colliculus under the conditions of a slow, progressive demyelination that is more reminiscent of MS. While demyelination and gliosis in the corpus callosum were observed, no significant changes occurred in any of the nuclei associated with the visual pathway, consistent with previous work by Taveggia et al. (2008). In addition, the temporal response of CPZ may be due to the differential expression profiles of oligodendrocytes in different CNS regions (Butt et al. 1995). For example, mice haplo-insufficient for type III neuregulin-1 (a growth factor that promotes oligodendrocyte and Schwann cell development) showed less myelination in the corpus callosum but no effect on the optic nerve and spinal cord, indicating regional differences in the regulation of oligodendrocyte function (Taveggia et al. 2008). Whether these factors (e.g. neuregulin-1 expression) lead to the heterogeneity of oligodendrocyte distribution in the brain vs spinal cord or in optic nerve (Ornelas et al. 2016), as well as in the response to CPZ, remains untested.

### Marked Proteomic Changes

Demyelination in the optic nerves of MS patients is inferred by changes in visual evoked potentials (Barton et al. 2019; Langwińska-Wośko et al. 2012; Leocani et al. 2018), although early changes (i.e. subtle alterations in vision) are not reported in the clinic until after an MS diagnosis has already been made. Increased abundance of inflammatory cytokines (e.g. tumour necrosis factor-α) and the axonal marker (neurofilament light chain) in the CSF have been proposed as early biomarkers to diagnose MS and/or optic neuritis in patients (Olesen et al. 2019). In addition, these markers could also then be considered as pre-symptomatic/early indicators of MS risk, since optic neuritis patients often go on to develop MS (Costello 2016; Kale 2016). However, there exist issues with these and other canonical proteins identified to date with regard to their capacity to serve as selective biomarkers for MS (Sen et al. 2021).

In this study, optic nerve/tract tissue was used to investigate whether CPZ-induced changes in the proteomic profile could lead to the identification of candidate proteoform biomarkers and/or some indication of initiating pathological mechanism(s). Top-down proteomic analysis revealed changes in the abundance of at least 75 proteoforms (Table 1) in the optic nerve/tract tissue, using stringent criteria to ensure only the most robust identifications. To understand the function of these proteoforms and their relevance to demyelinating CNS conditions such as MS or its animal models, a comprehensive literature search was carried out using the PubMed search engine. The identified proteome changes indicate a substantial similarity with those previously identified in EAE studies (Table 1). In part, this similarity is likely attributable to the large number (15) of proteomic studies carried out using the EAE model (Alt et al. 2005; Dagley et al. 2014; Farias et al. 2012; Fazeli et al. 2010, 2013; Gonzalez et al. 2019; Hasan et al. 2019; Jain et al. 2009, 2012; Jastorff et al. 2009; Linker et al. 2009; Liu et al. 2007; Raphael et al. 2017; Stoop et al. 2012; Vanheel et al. 2012), whereas only 6 studies used the CPZ model (Martin et al. 2018; Oveland et al. 2018; Partridge et al. 2016; Sen et al. 2019a; Szilagyi et al. 2020; Werner et al. 2010). Importantly, none of these studies investigated the optic nerve/tract proteome but used various other biological samples including the CSF, brain, spinal cord and tears. Thus, the current study is the first to investigate changes in the optic nerve/tract proteome. Although not directly comparable, the proteomic changes found here did in part overlap with previously identified changes in the abundance of certain canonical proteins; the major difference here was that the use of a top-down approach enabled resolution and identification of critical proteoforms. Thus, several of the proteoform changes in optic nerve/tract correlated with canonical protein changes previously seen in other samples, including the cerebellum (Hasan et al. 2019), spinal cord (Fazeli et al. 2013), CSF (Liu et al. 2009), tears (Salvisberg et al. 2014) and blood, reported in CPZ, EAE and MS studies (Berge et al. 2019; Partridge et al. 2016; Raphael et al. 2017). Notably, by separately analysing the soluble and membrane sub-proteomes and assessing proteoforms rather than canonical proteins, we have been able to gain more detailed information not available in previous studies. For example, neurofilament medium chain was found to increase in abundance in cerebral tissue from mice fed with 0.2% CPZ (Szilagyi et al. 2020) and the spinal cord from EAE mice (Farias et al. 2012; Hasan et al. 2019); the data here conclusively identified a decrease of one proteoform in the SP fraction of the optic nerve/tract tissue but a corresponding larger increase in a distinctly different proteoform in the MP sub-proteome (Table 1). While this clearly highlights the essential need to resolve and identify critical proteoforms rather than canonical protein sequences (Carbonara et al. 2021), as well as the potentially serious ramifications of not doing so, the findings may also indicate differential expression/abundance of neurofilament isoforms among different CNS regions, as likely already seen in the hippocampus and cortex (Mesulam and Geula 1991; Nakamura et al. 1992). Moreover, Szilagyi et al. (2020) fed 8-week male C57Bl/6 mice with 0.2% CPZ for 4 weeks (samples analysed at 12 weeks), whereas in the current study, mice were fed with 0.1% CPZ for 12 weeks, suggesting that prolonged feeding of CPZ may alter the abundance and localization of different neurofilament proteoforms. Furthermore, two other studies (Farias et al. 2012; Hasan et al. 2019) used spinal cord from EAE mice suggesting that differential disease induction (autoimmune in EAE vs metabolic changes in CPZ) may also alter the abundance of neurofilament proteoforms in the CNS. Notably, an increase of neurofilament light chain in serum has been considered as a potential pre-symptomatic biomarker of neurodegeneration in MS (Bjornevik et al. 2019; Varhaug et al. 2019); however, comparable changes also appear in other neurodegenerative disorders as well as in cases of neurotrauma raising questions as to the selectivity of this as a biomarker for MS, unless specific proteoforms are found to be altered in the different conditions (Sen et al. 2021).

Previous MS proteomic studies identified an increase in the abundance of heat shock protein 90-beta (HSP90β) in tears (Salvisberg et al. 2014) and in peripheral blood mononuclear cells (De Masi et al. 2009). However, neither of these studies provided evidence of full length, intact species. On the contrary, the current study identified a reduction of HSP90β in the optic nerve tissue. Perhaps, the increased abundance of heat shock protein in tears and the circulation is a breakdown product of HSP90β from other CNS regions such as the optic nerve. This may lead to a reduction in the abundance of HSP90β in the optic nerve as observed in the current study. Of note, HSP90β exerts two potential neuroprotective roles in the CNS tissue: firstly, it prevents protein misfolding and aggregation by its chaperone activity, and secondly, it inhibits multiple steps in the apoptosis process (Didonna and Opal 2019; Lanneau et al. 2007; Mosser and Morimoto 2004). In MS patients, these proteins (or, likely, proteoforms thereof) are overexpressed in neuronal cells and oligodendrocytes around demyelinated lesions, seemingly to protect these cells from degeneration (Cwiklinska et al. 2010; Turturici et al. 2014). Other possible reasons for the differences in the trends of protein abundance are the use of different analytical techniques, experimental model or sample analysed. For example, Szilagyi et al. (2020) and Hasan et al. (2019), respectively, used two different labelling variations of bottom-up proteomic analysis; one identified ~ 190 canonical proteins that appeared to change in abundance in the corpus callosum of CPZ-fed mice (Szilagyi et al. 2020), while the other identified ~ 1900 canonical protein changes in CNS samples from EAE mice (Hasan et al. 2019). In contrast, Farias et al. (2012) used a top-down (2DE) proteomic approach with spinal cords from EAE mice and identified alterations in 35 proteoforms (although only theoretical MW and *pI* were reported). Using tear samples from MS patients, Salvisberg et al. (2014) identified 42 canonical proteins differing in abundance between MS and control patients. Thus, as is often the case, bottom-up (i.e. shotgun) studies detected more apparent changes in the abundance of canonical proteins relative to proteome changes identified using a top-down (2DE) approach (De Masi et al. 2009; Farias et al. 2012; Hasan et al. 2019; Salvisberg et al. 2014; Szilagyi et al. 2020). Does this imply differences in sensitivity of the methods? This seems unlikely as bottom-up peptide analysis generally uses less stringent sequence coverage than top-down and only identifies canonical proteins by inference to amino acid sequences. Therefore, comparable to its correlate transcriptomics, the identification of a large number of potential canonical proteins is expected using the bottom-up method. In contrast, the top-down approach uses more stringent criteria to detect changes in intact *proteoforms* (i.e. a more selective analysis)*.* Therefore, top-down is likely to yield more reliable and focused data (i.e. changes in abundance of relevant species rather than total changes in a canonical protein sequence that likely represents many dozens of proteoforms) (Aebersold et al. 2018; Coorssen and Yergey 2015; Oliveira et al. 2014; Zhan et al. 2019). Thus, the likelihood of identifying a critical change relevant to underlying molecular mechanisms or the identification of a highly selective biomarker lies in the routine, high-resolution assessment of proteoforms (Sen et al. 2021).

Our previous detailed review of proteomic studies into MS found that at least nine proteoforms (of septin, tubulin, complement, glial fibrillary acidic protein, protein disulphide isomerase, calreticulin, hexokinase, aconitate hydratase and dynamin 1) consistently changed in abundance in both MS and animal models (Sen et al. 2021). Of these, five proteoforms (of septin, glial fibrillary acidic protein, aconitate hydratase, protein disulphide isomerase and tubulin) were also identified in the current study, suggesting that these may be potential early biomarkers. Opposite trends in abundance (i.e. increase or decrease) in different biological samples may also be attributed to the differential expression of proteins and distribution of proteoforms in these different samples, which depend on tissue function and the magnitude of pathological changes. For example, the spinal cords of EAE mice (i.e. the most pathologically affected CNS region in EAE) showed changes in the abundance of 1357 (uncategorised proteins were not considered) canonical proteins, whereas only ~ 50 protein changes were found in the brain stem and cerebellum (Hasan et al. 2019). The literature search was thus extended to include proteome changes in eye disorders (ED) to investigate their potential relevance to optic nerve/tract proteomic changes in CPZ-fed mice. Only three identifications (transitional endoplasmic reticulum ATPase, neurofilament light polypeptide and haemoglobin subunit alpha) were consistent with those reported in CSF samples from neuromyelitis optica (Bai et al. 2009) and optic neuritis (Olesen et al. 2019) and in tear samples from patients with dry eye disease (Jung et al. 2017). These findings suggest a link between eye diseases and the CPZ-induced optic nerve/tract proteome changes that requires further study, particularly with regard to likely early changes prior to an MS diagnosis.

### Aggregation and Oligomerization Proteoforms

The current study detected changes in 12 molecular chaperone proteoforms (Table 1). These changes are not unique to optic nerve/tract as they were reported previously in other CPZ (Partridge et al. 2016; Szilagyi et al. 2020), EAE (Hasan et al. 2019; Jain et al. 2012; Vanheel et al. 2012) and MS studies (De Masi et al. 2009; Salvisberg et al. 2014). Decreased molecular chaperone proteoforms have been linked to protein aggregation (Ciechanover and Kwon 2017; Liberek et al. 2008), which can contribute to neurodegeneration and demyelination associated with human degenerative diseases (David and Tayebi 2014; Soto and Pritzkow 2018). It has been shown that the increased expression of chaperones (e.g. heat shock proteins) in astrocytes and neurons inhibits apoptosis of these cells in rat spinal cord (Chang et al. 2014). Moreover, optic nerve regeneration and retinal ganglion cell survival in zebrafish were promoted by HSP70, and these processes were reduced when the HSP70 was inhibited (Nagashima et al. 2011). Another study demonstrated that neurite growth of rat retinal cells increased following the application of exogenous HSP αB-crystallin (Wang et al. 2012). Chaperones also modulate the cytoskeleton of neuronal cells and mediate their regeneration via enhancing intermediate filament assembly (Hirata et al. 2003). These observations indicate that the increased abundance of some chaperone proteoforms (e.g. HSP 70 kDa and HSP 47 kDa) in the present study could reflect the demands for these in the optic nerve/tract in order to reduce structural deformities and protect or regenerate neuronal tissue injured by CPZ exposure.

We (Sen et al. 2019a) and others (Liu et al. 2009) have found evidence of homo-oligomerization of proteoforms. The current data also suggest oligomerization (e.g. an approximate doubling of molecular weight) (Table 1) of some species such as tubulin alpha-1C chain (50.6 kDa monomer vs 110.6 kDa experimentally observed), serine protease inhibitor A3K (47.1 kDa vs 95.9 kDa), beta-synuclein (14.1 kDa vs 27.0 kDa) and charged multivesicular body protein 4b (24.9 kDa vs 58.4 kDa). While oligomerization of tubulin alpha-1C chain, serine protease inhibitor A3K, beta-synuclein or charged multivesicular body protein 4b have not previously been identified in CPZ-fed mice, earlier studies have observed that these proteins do indeed oligomerize (Carrell et al. 2008; Mozziconacci et al. 2008). Notable in the case of beta-synuclein is that it forms hetero-oligomers with alpha-synuclein, and these were found together in the ~ 27 kDa spot (SP12) (Table 1). Oligomerization of proteins can lead to protein aggregation which is argued to be the cause of many neurological diseases including MS (David and Tayebi 2014; Michaels et al. 2015). In addition, gel shifts in MW and *pI* relative to theoretical values (i.e. of the amino acid sequence only) were also observed for some of the identified species (Table 1, Fig. 3C) such as neurofilament medium polypeptide (96.0 kDa/4.6 vs 117.3 kDa/4.7), myelin-associated glycoprotein (70.1 kDa/4.9 vs 90.2 kDa/4.4), ATP synthase subunit alpha (59.9 kDa/9.6 vs 66.3 kDa/6.8) and cytochrome c oxidase subunit 5B (14.1 kDa/8.5 vs 17.5 kDa/5.9), consistent with post-translational modifications and thus the identification of select proteoforms (Rabilloud and Lelong 2011; Sen et al. 2019a).

### Structural Proteoforms

In the current study, major changes in cytoskeleton proteoforms such as actin and tubulin were detected in the optic nerve/tract. The alterations of these structural proteoforms in this slow progressive demyelinating model may reflect early changes in cellular structure as a result of CPZ-feeding. Importantly, the present study identified an increase in the abundance of intermediate filament proteins such as glial fibrillary acidic protein and vimentin, suggesting that astrocytes are activated in the optic nerve/tract (Sofroniew and Vinters 2010). However, histological examination of glial fibrillary acidic protein in the optic tract did not detect any significant difference in glial staining intensity relative to the Ctrl group. This suggests that 2DE is more sensitive in revealing early *proteoform* changes, perhaps due in part to the larger amount of sample analysed relative to tissue sections. These proteomic data are thus indicative of notable structural disturbances in the optic nerve/tract following CPZ-feeding that might contribute to conditions such as MS.

Notably, actin and tubulin are often used as ‘house-keeping’ loading controls in assays such as Western blotting, in the very risky and unfounded hope that they do not change under the conditions of the experiment (Zhang et al. 2012). The marked changes in abundance of these structural proteoforms in the current study argue strongly against this practice and are consistent with other reports cautioning against this (Eaton et al. 2013; Nie et al. 2017). Changes in the abundance of such inappropriately named ‘house-keeping’ proteins have also been shown in other proteomic studies, including CPZ (Sen et al. 2019a; Werner et al. 2010), EAE (Farias et al. 2012; Fazeli et al. 2010; Hasan et al. 2019) and MS (De Masi et al. 2009; Dumont et al. 2004; Hammack et al. 2004; Liu et al. 2009). Therefore, total protein concentrations must be assessed in each sample and equal concentrations used for analysis (Almuslehi et al. 2020; Hu et al. 2016; Noaman and Coorssen 2018; Sen et al. 2019a).

### Metabolic Proteoforms

Another key finding in this study was the detection of changes in the abundance of numerous proteoforms associated with metabolic and mitochondrial functions in the optic nerves/tracts. Metabolic dysregulation can lead to demyelination in the CPZ model (Caprariello et al. 2018; Sen et al. 2019a; Teo et al. 2021; Werner et al. 2010) and is thus hypothesized as an early dysfunction leading to MS. Despite finding changes in metabolic proteoforms, no demyelination was detected in the optic nerve/tract. This may be attributed to the highly compact myelin structure in the optic nerve tissue which increased the intensity of silver staining (i.e. resulting in saturation) and thus may have limited the detection of demyelination (Sen et al. 2020a). This interpretation is supported by observations in the diphtheria toxin model, in which alterations in axonal structure were observed by ultrastructural analysis despite no overt demyelination (Pohl et al. 2011). Likewise, reduction of myelin basic protein in the optic nerve may result from metabolic turnover (Namekata et al. 2014). Previously, we reported that ~ 50% of proteoforms that changed in abundance in whole brain samples were metabolic (Sen et al. 2019a), whereas here, in the optic nerve/tract, metabolic proteoforms constituted only ~ 24%. Albeit an indirect comparison, this suggests less metabolic disturbance in the optic nerve/tract tissue relative to the brain, and thus, demyelination is readily evident in the corpus callosum but not in the optic nerve/tract. Additional proteomic studies are thus required for direct comparison (optic nerve/tract vs corpus callosum) to investigate the threshold of changes in metabolic proteoforms that are necessary for demyelination in optic nerve.

It might be argued that the dosage of CPZ (e.g. 0.2 vs. 0.1%) plays a significant role in changing the profile of metabolic proteins. This seems less likely since a comparable level of demyelination occurred by feeding mice with 0.2% CPZ for 5 weeks or with 0.1% for 12 weeks (Sen et al. 2019a). Likewise, feeding mice with either 0.1% or 0.2% CPZ for 2 weeks yielded comparable demyelination and glial activation in the corpus callosum (Almuslehi et al. 2020). Therefore, it is expected that changes in the abundance of metabolic proteoforms in the optic nerve/tract following 0.1% CPZ-feeding for 12 weeks may be comparable to those seen in 0.2% CPZ-feeding for 5 weeks. Nonetheless, this does not rule out progressive but localized effects of CPZ in different areas of the CNS (including the optic nerve/tract). This study also detected changes in six proteoforms identified in our previous proteomic analysis of the brain (i.e. creatine kinase U-type, neurofilament light polypeptide, glial fibrillary acidic protein, ATP synthase subunit alpha, aconitate hydratase, charged multivesicular body protein 4b) (Sen et al. 2019a). Among these, only 3 (creatine kinase U-type, ATP synthase subunit alpha and aconitate hydratase) are recognized to be directly involved in metabolism. Creatine kinase exerts a variety of bioenergetic and neuroprotective properties in CNS and retinal neurons including buffering and stabilization of intracellular energy reserves, neutralizing calcium ion fluxes, inhibition of mitochondrial permeability and counteracting intracellular oxidative stress (Beal 2011; Sia et al. 2019). ATP synthase subunit expression is upregulated during ocular hypertension, and it is associated with increased ATP concentration in the retinal ganglion of rats (Kanamoto et al. 2019). An in vivo study showed that aconitate hydratase activity increased in optic nerve tissue 1 day following traumatic injury (Cummins et al. 2013). According to these observations, the increased abundance of creatine kinase U-type, ATP synthase subunit alpha and aconitate hydratase in the optic nerve/tract following CPZ-feeding is indicative of mitochondrial dysregulation. However, the main cluster of protein–protein interactions in our previous study was identified as metabolic (with malate dehydrogenase, succinate dehydrogenase, aspartate aminotransferase and oxoglutarate dehydrogenase protein (Sen et al. 2019a)), whereas these proteoforms were not identified in the current work. This suggests that minimal changes in the complement of proteoforms, and their interaction with key metabolic proteoforms (e.g. malate dehydrogenase, aconitate hydratase), are required to initiate metabolic disturbances that can induce downstream effects (e.g. demyelination of optic nerve/tract) in the CPZ-fed mice.

### Biological Functions and Interactions

Importantly, from the bioinformatic (UniProt, PANTHER and STRING) and literature (PubMed) analyses, complex linkages among identified proteoforms were indicated (Fig. 4). For example, the literature search linked proteoforms to diverse functions including structural, metabolic and axonal, suggesting CPZ-induced changes at multiple functional levels. Characterizing such potential interactions is important to understanding the underlying dysregulation of biological processes (Berggard et al. 2007; Sen et al. 2019a). Previous studies have shown that 50–80% of proteins undergo protein–protein interactions (Asgarov et al. 2021; Berggard et al. 2007; Dagley et al. 2014; Sen et al. 2019a). These observations are consistent with the idea that while proteins/proteoforms function as monomers, they also interact to form complexes in order to exert their molecular actions (Berggard et al. 2007; De Las Rivas and Fontanillo 2010; Keskin et al. 2016; Sen et al. 2019a). Consistent with these observations, the current data suggest that ~ 90% of the identified proteoforms were interconnected, indicating molecular cross-talk (Asgarov et al. 2021; Sen et al. 2019a). For example, myelin-associated glycoprotein, a molecule located in the axonal plasmalemma (inner aspect of the myelin sheath) of oligodendrocytes, is said to interact with axonal neurofilament microtubule proteins, leading to phosphorylation of neurofilament microtubules and modulation of axonal diameter (Nguyen et al. 2009). Another example is alpha-internexin, a neuronal protein implicated in neurodegenerative diseases, which cannot exert its effects independently but functions in association with other neurofilament proteins such as neurofilament medium chain, and this interaction is necessary for the axonal transport of neurofilament medium chain in CNS and optic nerve axons (Yuan et al. 2006). Overall, the protein–protein interaction analysis suggested that alterations in the abundance of the identified proteoforms in optic nerve/tract are likely interrupting molecular cross-talk among these species, thereby disrupting associated biochemical reactions and perhaps thereby contributing to disorders such as MS.

### Limitations and Future work

Despite our best efforts to minimize the experimental variables, we acknowledge certain drawbacks in the current study. Firstly, since 0.2% CPZ-feeding for 12 weeks can reduce visual function in mice (tested using multifocal electroretinograms (Namekata et al. 2014)), it would be important to correlate molecular changes with visual status in future studies. Secondly, this study relied on only one time point (i.e. 12 weeks) of CPZ-feeding. Therefore, future studies should use a temporal analysis to determine the earliest point at which proteoform changes occur as they may identify the triggers for the cascade of molecular alterations that we identified here after 12 weeks of slow demyelination. Finally, the bioinformatic analyses employed (e.g. PANTHER, STRING) are based primarily on literature reports concerning canonical proteins rather than specific proteoforms or oligomers, and this may also influence interpretation of the data in terms of canonical versus actual proteoform changes.

## Conclusions

This study was designed to investigate the effects on the visual pathway of a CPZ-feeding paradigm producing a slow, progressive demyelination, using both histological and proteomic analyses. While no significant histological changes were identified, the data established marked optic nerve/tract proteome dysregulation, which may assist in understanding molecular/cellular alterations that are linked to demyelinating conditions. The data highlight the importance of using top-down proteomic analyses in resolving key early proteoform changes as these seem likely to precede demyelination and glial activation. Furthermore, this detailed investigation (histology, proteomics, bioinformatics and literature mining) provides further insight into the regionally differential effects of CPZ on the CNS. Additionally, the current dataset serves as a baseline for changes in the optic nerve/tract proteome of the CPZ model, in support of future studies.

## Supplementary Information

Below is the link to the electronic supplementary material.

Supplementary figure 1 file1 (JPG 4 MB)

Supplementary Figure 1 legend file2 (DOCX 16 KB)

Supplementary figure 2 file3 (JPG 3 MB)

Supplementary Figure 2 legend file4 (DOCX 13 KB)

Supplementary figure 3 file5 (JPG 1 MB)

Supplementary Figure 3 legend file6 (DOCX 12 KB)

Supplementary figure 4 file7 (JPG 2 MB)

Supplementary Figure 4 legend file8 (DOCX 12 KB)

Supplementary Table 1 file9 (DOCX 42 KB)

## Data Availability

All data generated or analysed during this study are provided inside the manuscript and electronic supplementary information files.
