# Supplementary figures and images for: Histological and Top-Down Proteomic Analyses of the Visual Pathway in the Cuprizone Demyelination Model
Source: J Mol Neurosci. 2022 May 30;72(6):1374–401. doi: 10.1007/s12031-022-01997-w (PMC9170674; doi:10.1007/s12031-022-01997-w)

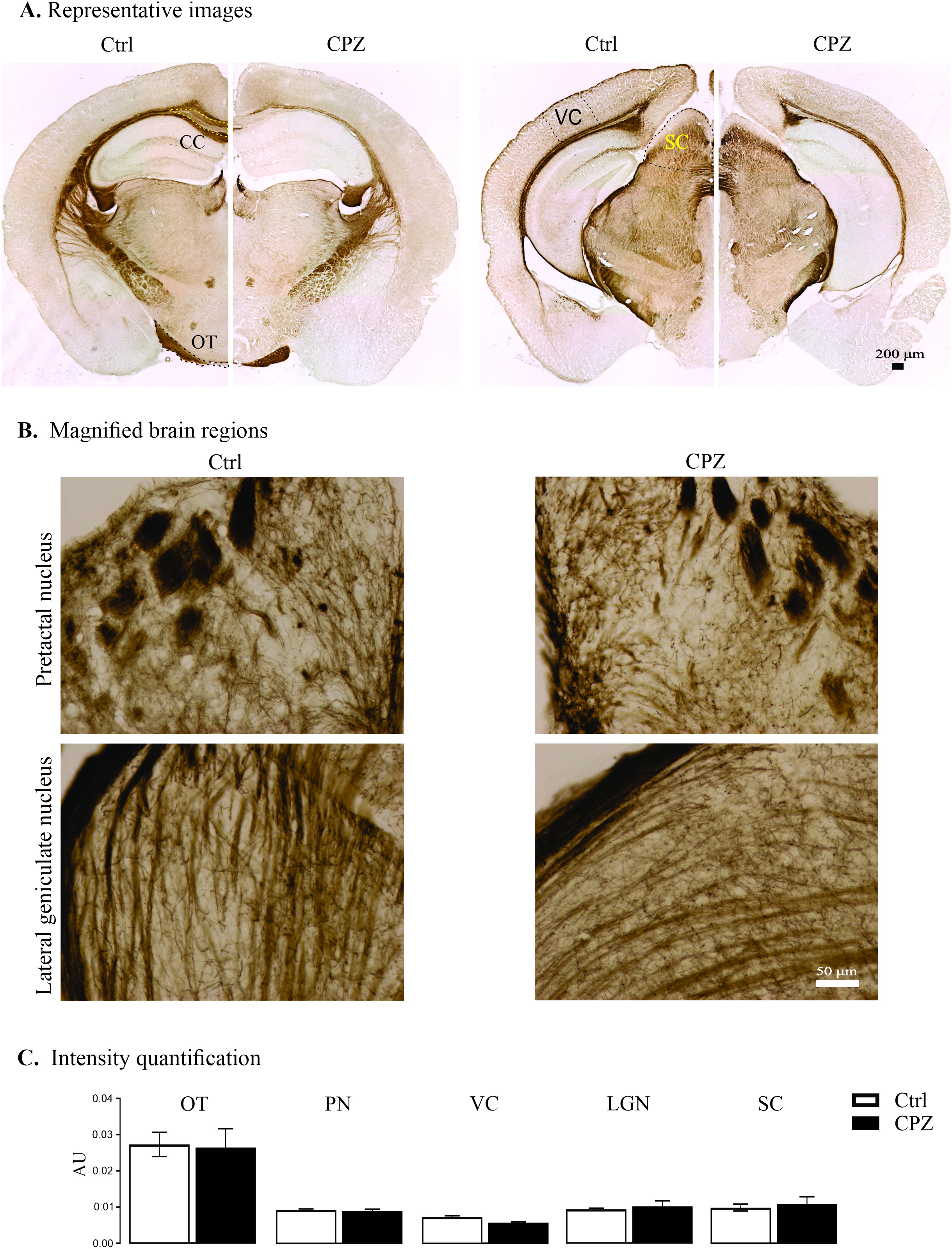

Supplement: Supplementary file 1 — Supplementary figure 1 file1 (JPG 4 MB) [file 12031_2022_1997_MOESM1_ESM.jpg]

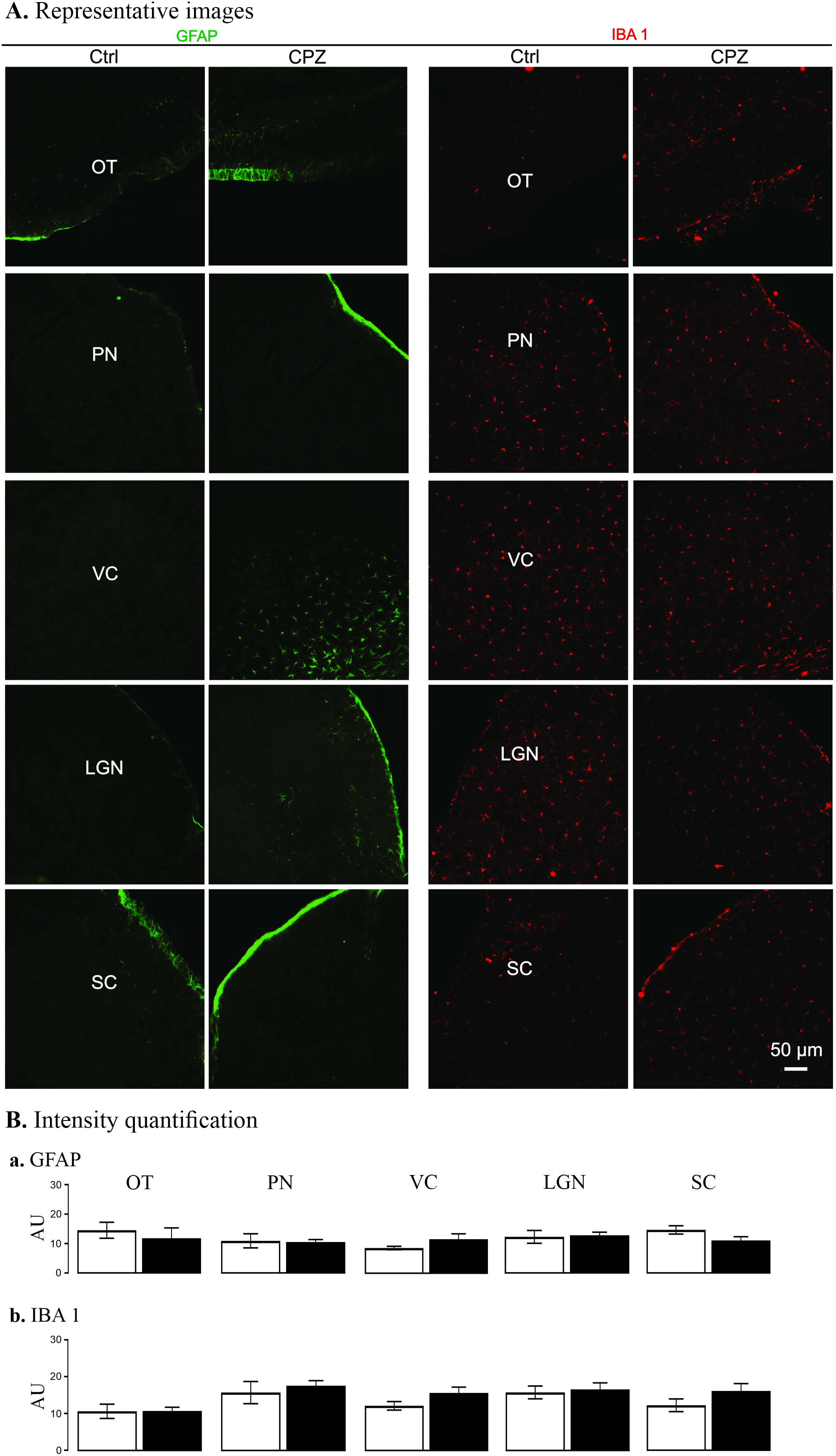

Supplement: Supplementary file 3 — Supplementary figure 2 file3 (JPG 3 MB) [file 12031_2022_1997_MOESM3_ESM.jpg]

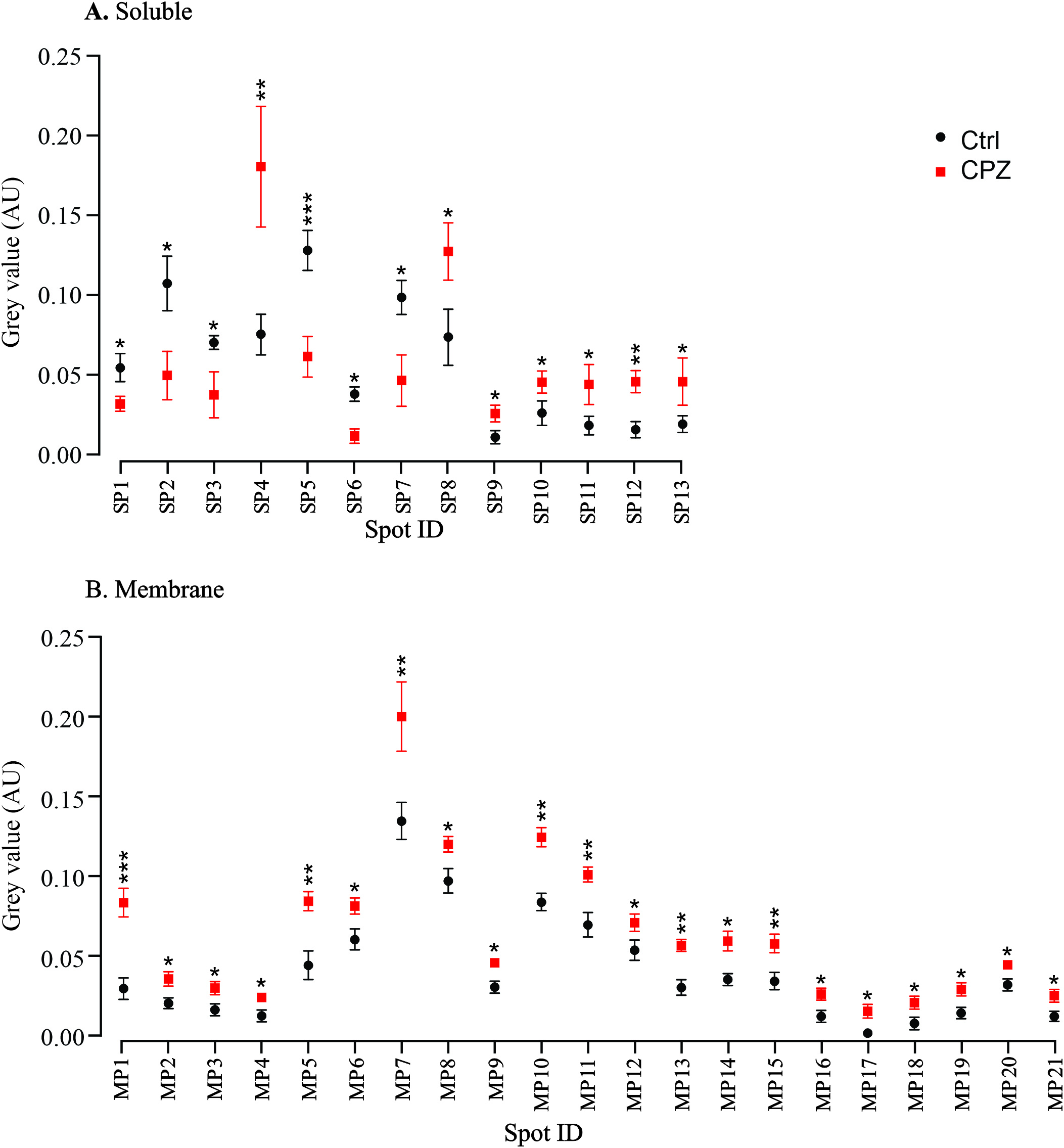

Supplement: Supplementary file 5 — Supplementary figure 3 file5 (JPG 1 MB) [file 12031_2022_1997_MOESM5_ESM.jpg]

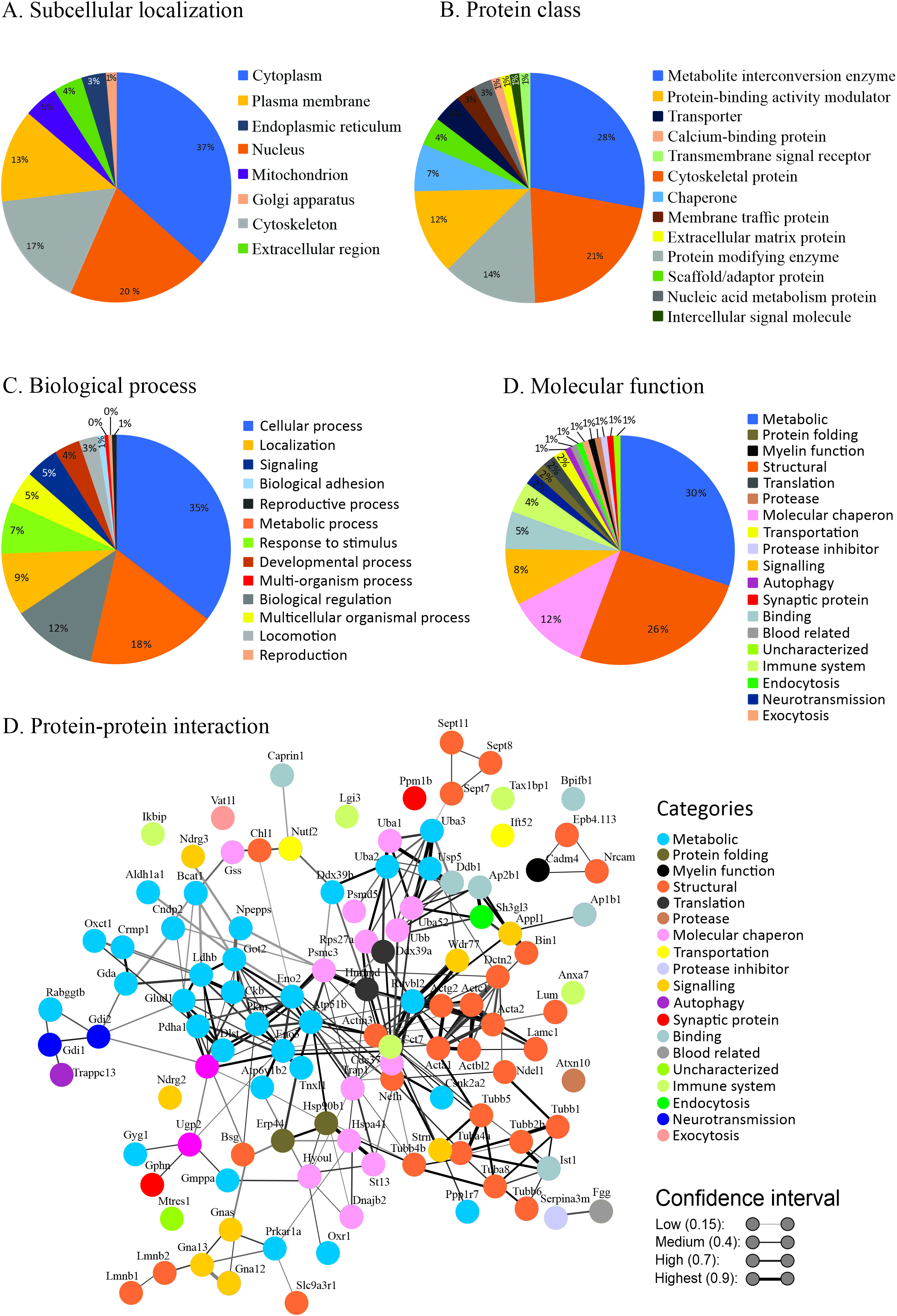

Supplement: Supplementary file 7 — Supplementary figure 4 file7 (JPG 2 MB) [file 12031_2022_1997_MOESM7_ESM.jpg]
