# Supplementary material for: Histological and Top-Down Proteomic Analyses of the Visual Pathway in the Cuprizone Demyelination Model
Source: J Mol Neurosci. 2022 May 30;72(6):1374–401. doi: 10.1007/s12031-022-01997-w (PMC9170674; doi:10.1007/s12031-022-01997-w)
Supplement: Supplementary file 2 — Supplementary Figure 1 legend file2 (DOCX 16 KB) [file 12031_2022_1997_MOESM2_ESM.docx]

**Supplementary Figure 1**: **A**) Representative images of silver-stained whole brain coronal sections and the regions of interest from Ctrl and CPZ-fed mice. **B**) Magnified silver-stained images from the pretactal nucleus and lateral geniculate nucleus. **C**) Quantification of staining (arbitrary unit, AU); silver staining (n=5 sections/animal, 5 animals/group) showed no changes after CPZ-feeding in the following brain regions: OT, optic tract; PN, pretectal nucleus; VC, visual cortex; LGN, lateral geniculate nucleus and SC, superior colliculus. Data are presented as mean ± SEM. A two-tailed t-test was used to determine differences between groups (*p<0.05).
