# Supplementary material for: Histological and Top-Down Proteomic Analyses of the Visual Pathway in the Cuprizone Demyelination Model
Source: J Mol Neurosci. 2022 May 30;72(6):1374–401. doi: 10.1007/s12031-022-01997-w (PMC9170674; doi:10.1007/s12031-022-01997-w)
Supplement: Supplementary file 4 — Supplementary Figure 2 legend file4 (DOCX 13 KB) [file 12031_2022_1997_MOESM4_ESM.docx]

**Supplementary Figure 2**: Representative images of GFAP positive astrocytes and IBA 1 positive microglia in visual pathways (3-5 sections/animal, 3-5 animals/group). Abbreviations: OT, optic tract; PN, pretectal nucleus; VC, visual cortex; LGN, lateral geniculate nucleus and SC, superior colliculus.
