# Supplementary material for: Histological and Top-Down Proteomic Analyses of the Visual Pathway in the Cuprizone Demyelination Model
Source: J Mol Neurosci. 2022 May 30;72(6):1374–401. doi: 10.1007/s12031-022-01997-w (PMC9170674; doi:10.1007/s12031-022-01997-w)
Supplement: Supplementary file 6 — Supplementary Figure 3 legend file6 (DOCX 12 KB) [file 12031_2022_1997_MOESM6_ESM.docx]

**Supplementary Figure 3**: **Quantification of the raw grey values of each significant change in spot volume.**

**A**) shows the changes in the abundance of identified spots in gels of the soluble sub-proteome from CPZ-fed mice compared to the corresponding spots in the Ctrl gels. Of 13 spots, 7 were significantly (p<0.05) increased and 6 decreased. **B**) shows an increase in abundance of all spots found to change significantly (p<0.05) in volume in gels of the membrane sub-proteome compared to the corresponding spots in the Ctrl gels. Asterisks indicate the followings: *p<0.05, **p<0.01, ***p<0.001.
