# Supplementary material for: Histological and Top-Down Proteomic Analyses of the Visual Pathway in the Cuprizone Demyelination Model
Source: J Mol Neurosci. 2022 May 30;72(6):1374–401. doi: 10.1007/s12031-022-01997-w (PMC9170674; doi:10.1007/s12031-022-01997-w)
Supplement: Supplementary file 8 — Supplementary Figure 4 legend file8 (DOCX 12 KB) [file 12031_2022_1997_MOESM8_ESM.docx]

**Supplementary Figure 4: Functional clustering and protein–protein interactions of the identified optic nerve proteoforms in Supplementary Table 1.**

Pie charts show the distribution of identified proteoforms according to (**A**) Subcellular localizations (characterized using UniProt), (**B**) Protein classes (categorized using PANTHER), (**C**) Biological processes (categorized using PANTHER), and (**D**) Molecular functions (via literature search). (**E**) Protein–protein interactions association network maps. The strength of interactions is indicated by the thickness of the lines. The colour coding of proteins in the STRING is based on the molecular functions (**D**).
