# Supplementary material for: Histological and Top-Down Proteomic Analyses of the Visual Pathway in the Cuprizone Demyelination Model
Source: J Mol Neurosci. 2022 May 30;72(6):1374–401. doi: 10.1007/s12031-022-01997-w (PMC9170674; doi:10.1007/s12031-022-01997-w)
Supplement: Supplementary file 9 — Supplementary Table 1 file9 (DOCX 42 KB) [file 12031_2022_1997_MOESM9_ESM.docx]

| **Supplementary Table 1. Additional identified optic nerve proteoforms** | | | | | | | | | | |
| --- | --- | --- | --- | --- | --- | --- | --- | --- | --- | --- |
| **Spot ID** | **Response to CPZ (ratio/p-value)** | **Gene**  **ID** | **Identified proteoform** | **Protein accession** | **Theoretical MW (KD)/*pI*** | **Observed MW (KD)/*pI*** | **PLG**  **Score** | **Sequence coverage (%)** | **Matched peptides** |  |
|  |  |  |  |  |  |  |  |  |  |  |
| SP1 | (1.57/ 0.049)↓ | Gdi1 | Rab GDP dissociation inhibitor alpha | P50396 | 51.1/4.8 | 117.3/4.7 | 898.2 | 63.5 | 33 |  |
|  |  | Trap1 | Heat shock protein 75 kDa_ mitochondrial | Q9CQN1 | 80.6/6.3 |  | 384.9 | 15.3 | 7 |  |
|  |  | Nrcam | Neuronal cell adhesion molecule | Q810U4 | 139.4/5.5 |  | 210.3 | 21.3 | 18 |  |
|  |  | Gdi2 | Rab GDP dissociation inhibitor beta | Q61598 | 51.1/4.8 |  | 181.1 | 24.0 | 8 |  |
|  |  | Usp5 | Ubiquitin carboxyl-terminal hydrolase 5 | P56399 | 96.7/4.5 |  | 166.3 | 21.0 | 13 |  |
|  |  | Bpifb1 | BPI fold-containing family B member 1 | Q61114 | 52.6/6.0 |  | 131.8 | 11.0 | 6 |  |
|  |  | Chl1 | Neural cell adhesion molecule L1-like protein | P70232 | 136.2/5.5 |  | 110.0 | 13.6 | 17 |  |
| SP2 | (3.58/  0.002)↓ | Acta2 | Actin_ aortic smooth muscle | P62737 | 42.4/5.1 | 110.6/4.9 | 869.1 | 32.1 | 18 |  |
|  |  | Actg2 | Actin_ gamma-enteric smooth muscle | P63268 | 42.3/5.2 |  | 869.1 | 32.1 | 18 |  |
|  |  | Acta1 | Actin_ alpha skeletal muscle | P68134 | 42.4/5.1 |  | 869.1 | 32.1 | 18 |  |
|  |  | Actc1 | Actin_ alpha cardiac muscle 1 | P68033 | 42.4/5.1 |  | 869.1 | 32.1 | 18 |  |
|  |  | Actbl2 | Beta-actin-like protein 2 | Q8BFZ3 | 42.3/5.2 |  | 470.9 | 12.5 | 8 |  |
|  |  | Tubb1 | Tubulin beta-1 chain | A2AQ07 | 51.1/4.8 |  | 232.3 | 20.8 | 9 |  |
|  |  | Ldhb | L-lactate dehydrogenase B chain | P16125 | 36.9/5.7 |  | 189.8 | 18.0 | 5 |  |
|  |  | Rps27a | Ubiquitin-40S ribosomal protein S27a | P62983 | 18.3/10.2 |  | 131.6 | 16.0 | 4 |  |
|  |  | Ubb | Polyubiquitin-B | P0CG49 | 34.4/7.8 |  | 126.9 | 8.2 | 3 |  |
|  |  | Uba52 | Ubiquitin-60S ribosomal protein L40 | P62984 | 15.0/10.5 |  | 126.9 | 19.5 | 3 |  |
| SP3 | (2.25/  0.05)↓ | Serpina3m | Serine protease inhibitor A3M | Q03734 | 47.2/5.7 | 95.9/  4.6 | 317.7 | 5.5 | 5 |  |
| SP4 | (1.67/  0.001)↑ | Ddb1 | DNA damage-binding protein 1 | Q3U1J4 | 128.1/5.0 | 93.5/  5.2 | 1180.4 | 29.4 | 36 |  |
|  |  | Oxr1 | Oxidation resistance protein 1 | Q4KMM3 | 96.4/5.1 |  | 623.9 | 29.5 | 25 |  |
|  |  | Hyou1 | Hypoxia up-regulated protein 1 | Q9JKR6 | 111.4/4.9 |  | 346.3 | 22.0 | 25 |  |
|  |  | Uba1 | Ubiquitin-like modifier-activating enzyme 1 | Q02053 | 119.0/5.3 |  | 345.6 | 22.1 | 23 |  |
| SP5 | (3.12/  0.001)↓ | Ap2b1 | AP-2 complex subunit beta | Q9DBG3 | 105.5/5.1 | 90.8/  5.1 | 386.4 | 19.7 | 20 |  |
|  |  | Hsp90b1 | Endoplasmin | P08113 | 92.8/4.6 |  | 339.9 | 25.8 | 22 |  |
|  |  | Tax1bp1 | Tax1-binding protein 1 homolog | Q3UKC1 | 94.7/5.1 |  | 275.5 | 22.7 | 21 |  |
|  |  | Strn | Striatin | O55106 | 86.4/5.0 |  | 175.9 | 20.8 | 15 |  |
|  |  | Hspa4l | Heat shock 70 kDa protein 4L | P48722 | 95.2/5.4 |  | 169.3 | 9.7 | 11 |  |
|  |  | Ap1b1 | AP-1 complex subunit beta-1 | O35643 | 104.8/4.9 |  | 168.1 | 11.7 | 13 |  |
|  |  | Caprin1 | Caprin-1 | Q60865 | 78.3/5.0 |  | 142.2 | 7.2 | 7 |  |
| SP6 | (2.01/  0.038)↓ | − | − | − | − | 90.2/  4.4 | − | − | − |  |
| SP7 | (1.59/  0.021)↓ | Npepps | Puromycin-sensitive aminopeptidase | Q11011 | 104.0/5.5 | 90.1/  5.2 | 448.2 | 22.1 | 23 |  |
|  |  | Actn3 | Alpha-actinin-3 | O88990 | 103.7/5.1 |  | 267.9 | 5.1 | 6 |  |
|  |  | Gphn | Gephyrin | Q8BUV3 | 84.0/5.2 |  | 234.7 | 12.4 | 9 |  |
|  |  | Uba2 | SUMO-activating enzyme subunit 2 | Q9Z1F9 | 71.3/4.9 |  | 192.2 | 8.5 | 9 |  |
| SP8 | (1.67/  0.041)↑ | − | − | − | − | 87.7/  6.8 | − | − | − |  |
| SP9 | (1.72/  0.035)↑ | − | − | − | − | 85.5/  6.9 | − | − | − |  |
| SP10 | (1.69/  0.037)↑ | − | − | − | − | 77.2/  8.5 | − | − | − |  |
| SP11 | (1.69/  0.015)↑ | Txnl1 | Thioredoxin-like protein 1 | Q8CDN6 | 32.6/4.6 | 58.4/  4.7 | 585.4 | 18.3 | 4 |  |
|  |  | Rabggtb | Geranylgeranyl transferase type-2 subunit beta | P53612 | 38.5/4.8 |  | 354.2 | 14.5 | 5 |  |
|  |  | Dnajb2 | DnaJ homolog subfamily B member 2 | Q9QYI5 | 35.7/5.6 |  | 352.1 | 10.55 | 4 |  |
| SP12 | (2.29/  0.015)↑ | − | − | − | − | 27.0/  4.6 | − | − | − |  |
| SP13 | (1.61/  0.04)↑ | Nutf2 | Nuclear transport factor 2 | P61971 | 14.6/5.0 | 17.6/  4.9 | 625.4 | 29.1 | 4 |  |
| MP1 | (2.75/  0.01)↑ | Tuba4a | Tubulin alpha-4A chain | P68368 | 50.7/4.8 | 132.3/  4.7 | 509.0 | 15.9 | 6 |  |
|  |  | Nefh | Neurofilament heavy polypeptide | P19246 | 117.3/5.6 |  | 283.9 | 13.0 | 15 |  |
|  |  | Tubb2b | Tubulin beta-2B chain | Q9CWF2 | 50.4/4.6 |  | 208.2 | 24.7 | 13 |  |
|  |  | Tubb6 | Tubulin beta-6 chain | Q922F4 | 50.5/4.6 |  | 125.5 | 24.6 | 12 |  |
|  |  | Tuba8 | Tubulin alpha-8 chain | Q9JJZ2 | 50.7/4.8 |  | 121.3 | 9.8 | 3 |  |
|  |  | Tubb4b | Tubulin beta-4B chain | P68372 | 50.3/4.6 |  | 109.4 | 20.9 | 11 |  |
|  |  | Tubb5 | Tubulin beta-5 chain | P99024 | 50.1/4.6 |  | 107.2 | 17.6 | 9 |  |
|  |  | Lamc1 | Laminin subunit gamma-1 | P02468 | 182.9/4.9 |  | 104.5 | 5.5 | 10 |  |
| MP2 | (3.55/  0.01)↑ | − | − | − | − | 97.8/  4.6 | − | − | − |  |
| MP3 | (1.63/  0.03)↑ | − | − | − | − | 90.1/  6.9 | − | − | − |  |
| MP4 | (2.19/  0.03)↑ | − | − | − | − | 82.8/  6.8 | − | − | − |  |
| MP5 | (1.84/  0.02)↑ | Glud1 | Glutamate dehydrogenase 1_ mitochondrial | P26443 | 61.7/8.0 | 66.4/  6.8 | 353.6121 | 23.1 | 10 |  |
|  |  | Pkm | Pyruvate kinase PKM | P52480 | 58.4/7.2 |  | 310.0 | 13.8 | 5 |  |
|  |  | Ugp2 | UTP-glucose-1-phosphate uridylyl transferase | Q91ZJ5 | 57.2/7.5 |  | 280.8 | 21.3 | 10 |  |
| MP6 | (1.71/  0.02)↑ | Oxct1 | Succinyl-CoA:3-ketoacid coenzyme A transferase 1_ mitochondrial | Q9D0K2 | 56.4/8.7 | 64.1/  6.7 | 180.9 | 7.7 | 4 |  |
|  |  | Cct7 | T-complex protein 1 subunit eta | P80313 | 60.2/7.8 |  | 167.7 | 25.6 | 17 |  |
|  |  | Aldh1a1 | Retinal dehydrogenase 1 | P24549 | 55.1/7.7 |  | 127.4 | 9.6 | 6 |  |
|  |  | Lgi3 | Leucine-rich repeat LGI family member 3 | Q8K406 | 62.6/8.1 |  | 101.0 | 9.1 | 5 |  |
| MP7 | (1.51/ | − | − | − | − | 57.7/  4.8 | − | − | − |  |
| MP8 | (1.56/ | − | − | − | − | 55.9/  4.9 | − | − | − |  |
| MP9 | (1.66/  0.004)↑ | Dctn2 | Dynactin subunit 2 | Q99KJ8 | 44.2/5.0 | 53.0/  4.7 | 7940.9 | 62.2 | 27 |  |
|  |  | Psmd5 | 26S proteasome non-ATPase regulatory subunit 5 | Q8BJY1 | 56.4/4.9 |  | 6832.0 | 38.7 | 19 |  |
|  |  | Eno2 | Gamma-enolase | P17183 | 47.6/4.8 |  | 3684.0 | 54.8 | 26 |  |
|  |  | Atxn10 | Ataxin-10 | P28658 | 54.3/5.0 |  | 3042.0 | 42.7 | 23 |  |
|  |  | Psmc3 | 26S proteasome regulatory subunit 6A | O88685 | 49.8/4.9 |  | 2479.1 | 59.1 | 33 |  |
|  |  | Vat1l | Synaptic vesicle membrane protein VAT-1 homolog-like | Q80TB8 | 46.2/4.8 |  | 1626.5 | 25.2 | 10 |  |
|  |  | Bin1 | Myc box-dependent-interacting protein 1 | O08539 | 64.7/4.8 |  | 1600.1 | 33.2 | 16 |  |
|  |  | Lum | Lumican | P51885 | 38.7/6.0 |  | 1528.2 | 20.7 | 9 |  |
|  |  | Uba3 | NEDD8-activating enzyme E1 catalytic subunit | Q8C878 | 52.4/5.2 |  | 1351.5 | 23.4 | 10 |  |
|  |  | Ist1 | IST1 homolog | Q9CX00 | 39.6/5.1 |  | 1125.2 | 13.3 | 6 |  |
|  |  | Cadm4 | Cell adhesion molecule 4 | Q8R464 | 43.2/5.9 |  | 1042.7 | 19.6 | 7 |  |
|  |  | Eno1 | Alpha-enolase | P17182 | 47.5/6.4 |  | 772.4 | 18.2 | 4 |  |
|  |  | Eno3 | Beta-enolase | P21550 | 47.4/6.8 |  | 755.5 | 7.8 | 4 |  |
|  |  | Anxa7 | Annexin A7 | Q07076 | 50.2/5.0 |  | 735.1 | 21.2 | 10 |  |
|  |  | Cdc37 | Hsp90 co-chaperone Cdc37 | Q61081 | 45.1/5.1 |  | 425.7 | 30.1 | 11 |  |
|  |  | Ift52 | Intra-flagellar transport protein 52 homolog | Q62559 | 48.6/4.9 |  | 400.9 | 12.9 | 6 |  |
|  |  | Erp44 | Endoplasmic reticulum resident protein 44 | Q9D1Q6 | 47.3/4.9 |  | 381.7 | 20.2 | 8 |  |
|  |  | Appl1 | DCC-interacting protein 13-alpha | Q8K3H0 | 80.0/5.1 |  | 321.8 | 22.9 | 11 |  |
| MP10 | (1.52/  0.001)↑ | St13 | Hsc70-interacting protein | Q99L47 | 41.8/5.0 | 51.2/  4.8 | 2535.6 | 32.9 | 14 |  |
|  |  | Gda | Guanine deaminase | Q9R111 | 51.5/5.2 |  | 2095.4 | 23.1 | 11 |  |
|  |  | Slc9a3r1 | Na(+)/H(+) exchange regulatory cofactor NHE-RF1 | P70441 | 38.9/5.6 |  | 2005.0 | 47.9 | 14 |  |
|  |  | Ruvbl2 | RuvB-like 2 | Q9WTM5 | 51.3/5.3 |  | 1916.1 | 36.1 | 14 |  |
|  |  | Septin8 | Septin-8 | Q8CHH9 | 50.2/5.6 |  | 1819.1 | 18.4 | 8 |  |
|  |  | Fgg | Fibrinogen gamma chain | Q8VCM7 | 50.1/5.5 |  | 1786.2 | 32.3 | 14 |  |
|  |  | Ddx39b | Spliceosome RNA helicase Ddx39b | Q9Z1N5 | 49.5/5.3 |  | 1564.6 | 16.1 | 7 |  |
|  |  | Gss | Glutathione synthetase | P51855 | 52.5/5.5 |  | 1546.3 | 27.4 | 14 |  |
|  |  | Ckb | Creatine kinase B-type | Q04447 | 43.0/5.3 |  | 1111.4 | 14.4 | 4 |  |
|  |  | Atp5f1b | ATP synthase subunit beta_ mitochondrial | P56480 | 56.3/5.0 |  | 1043.1 | 34.8 | 12 |  |
|  |  | Gnas | Guanine nucleotide-binding protein G(s) subunit alpha isoforms XLas | Q6R0H7 | 122.6/4.5 |  | 1019.7 | 10.6 | 10 |  |
|  |  | Gnas | Guanine nucleotide-binding protein G(s) subunit alpha isoforms short | P63094 | 46.1/5.4 |  | 1010.0 | 20.6 | 7 |  |
|  |  | Septin11 | Septin-11 | Q8C1B7 | 50.0/6.3 |  | 789.0 | 20.4 | 9 |  |
|  |  | Ddx39a | ATP-dependent RNA helicase DDX39A | Q8VDW0 | 49.6/5.3 |  | 729.1 | 15.7 | 6 |  |
|  |  | Septin7 | Septin-7 | O55131 | 50.9/8.8 |  | 713.1 | 14.5 | 5 |  |
|  |  | Gna13 | Guanine nucleotide-binding protein subunit alpha-13 | P27601 | 44.4/8.3 |  | 699.1 | 10.9 | 4 |  |
|  |  | Prkar1a | cAMP-dependent protein kinase type I-alpha regulatory subunit | Q9DBC7 | 43.5/5.1 |  | 321.1 | 20.2 | 7 |  |
|  |  | Dlst | Dihydrolipoyllysine-residue succinyltransferase component of 2-oxoglutarate dehydrogenase complex_ mitochondrial | Q9D2G2 | 49.33/9.4 |  | 286.4 | 9.9 | 7 |  |
|  |  | Trappc13 | Trafficking protein particle complex subunit 13 | Q3TIR1 | 47.2/5.2 |  | 267.6 | 8.4 | 6 |  |
|  |  | Cndp2 | Cytosolic non-specific dipeptidase | Q9D1A2 | 53.2/5.3 |  | 239.0 | 14.7 | 6 |  |
|  |  | Atp6v1b2 | V-type proton ATPase subunit B_ brain isoform | P62814 | 56.9/5.5 |  | 223.6 | 8.8 | 4 |  |
|  |  | Mtres1 | Uncharacterized protein C6orf203 homolog | Q9CQF4 | 28.0/9.9 |  | 168.0 | 30 | 5 |  |
|  |  | Lmnb2 | Lamin-B2 | P21619 | 67.4/5.3 |  | 162.2 | 13.1 | 11 |  |
| MP11 | (1.86/  0.007)↑ | Epb41l3  Bsg | Band 4.1-like protein 3 | Q9WV92 | 103.8/5.0 | 50.6/  7.0 | 109.5 | 9.6 | 15 |  |
|  |  |  | Basigin | P18572 | 42.9/5.5 |  | 106.4 | 5.9 | 3 |  |
| MP12 | (1.64/  0.02)↑ | Ndrg3 | Protein NDRG3 | Q9QYF9 | 42.1/4.9 | 49.1/  6.6 | 531.8 | 13.3 | 5 |  |
|  |  | Ndrg2 | Protein NDRG2 | Q9QYG0 | 41.1/5.1 |  | 458.4 | 24.3 | 9 |  |
|  |  | Ndel1 | Nuclear distribution protein nudE-like 1 | Q9ERR1 | 38.6/5.0 |  | 449.2 | 23.8 | 8 |  |
|  |  | Wdr77 | Methylosome protein 50 | Q99J09 | 37.6/4.9 |  | 439.3 | 17.6 | 5 |  |
|  |  | Sh3gl3 | Endophilin-A3 | Q62421 | 39.1/4.9 |  | 370.5 | 25.1 | 8 |  |
|  |  | Lmnb1 | Lamin-B1 | P14733 | 67.0/4.9 |  | 359.7 | 32.8 | 21 |  |
|  |  | Gyg1 | Glycogenin-1 | Q9R062 | 37.6/4.9 |  | 239.8 | 5.1 | 3 |  |
|  |  | Ikbip | Inhibitor of nuclear factor kappa-B kinase-interacting protein | Q9DBZ1 | 42.6/4.8 |  | 171.8 | 14.2 | 5 |  |
|  |  | Ppp1r7 | Protein phosphatase 1 regulatory subunit 7 | Q3UM45 | 41.4/4.7 |  | 164.3 | 16.6 | 8 |  |
|  |  | Ppm1b | Protein phosphatase 1B | P36993 | 43.4/4.9 |  | 114.5 | 25.4 | 10 |  |
|  |  | Crmp1 | Dihydropyrimidinase-related protein 1 | P97427 | 62.5/6.0 |  | 101.3 | 8.7 | 6 |  |
| MP13 | (2.19/  0.001)↑ | Got2 | Aspartate aminotransferase_ mitochondrial | P05202 | 47.8/9.4 | 48.5/  6.6 | 279.6311 | 13.7 | 7 |  |
| MP14 | (2.07/  0.02)↑ | Pdha1 | Pyruvate dehydrogenase E1 component subunit alpha_ somatic form_ mitochondrial | P35486 | 43.9/8.1 | 48.1/  6.8 | 555.5 | 11.8 | 6 |  |
|  |  | Hnrnpd | Heterogeneous nuclear ribonucleoprotein D0 | Q60668 | 38.5/7.9 |  | 237.8 | 6.2 | 3 |  |
| MP15 | (1.68/  0.001)↑ | Gmppa | Mannose-1-phosphate guanyltransferase alpha | Q922H4 | 46.6/7.4 | 46.3/  4.8 | 253.8 | 8.33 | 3 |  |
|  |  | Csnk2a2 | Casein kinase II subunit alpha | O54833 | 41.4/8.8 |  | 208.1 | 6.6 | 4 |  |
| MP16 | (1.83/  0.005)↑ | − | − | − | − | 43.9/  4.0 | − | − | − |  |
| MP17 | (6.07/  0.03)↑ | Bcat1 | Branched-chain-amino-acid aminotransferase_ cytosolic | P24288 | 43.4/5.1 | 41.5/  4.4 | 177.7 | 5.4 | 3 |  |
|  |  | Gna12 | Guanine nucleotide-binding protein subunit alpha-12 | P27600 | 44.3/10.5 |  | 138.0 | 8.7 | 4 |  |
| MP18 | (1.66/  0.001)↑ | − | − | − | − | 40.9/  4.6 | − | − | − |  |
| MP19 | (2.21/  0.007)↑ | − | − | − | − | 21.5/  4.9 | − | − | − |  |
| MP20 | (1.56/  0.007)↑ | − | − | − | − | 17.5/  5.9 | − | − | − |  |
| MP21 | (1.65/  0.01)↑ | − | − | − | − | 11.2/  6.7 | − | − | − |  |
